# Application of the CRISPR/Cas9-based gene editing technique in basic research, diagnosis, and therapy of cancer

**DOI:** 10.1186/s12943-021-01431-6

**Published:** 2021-10-01

**Authors:** Huimin Zhang, Chunhong Qin, Changming An, Xiwang Zheng, Shuxin Wen, Wenjie Chen, Xianfang Liu, Zhenghua Lv, Pingchang Yang, Wei Xu, Wei Gao, Yongyan Wu

**Affiliations:** 1 Shanxi Key Laboratory of Otorhinolaryngology Head and Neck Cancer, Shanxi Province Clinical Medical Research Center for Precision Medicine of Head and Neck Cancer, Department of Otolaryngology Head & Neck Surgery, First Hospital of Shanxi Medical University, Taiyuan, 030001 Shanxi China; 2 Department of Biochemistry & Molecular Biology, Shanxi Medical University, Taiyuan, 030001 Shanxi China; 3 Department of Head and Neck Surgery, National Cancer Center/National Clinical Research Center for Cancer/Cancer Hospital, Chinese Academy of Medical Sciences and Peking Union Medical College, Beijing, 100021 China; 4 General Hospital, Clinical Medical Academy, Shenzhen University, Shenzhen, 518055 Guangdong China; 5 Department of Otolaryngology Head & Neck Surgery, Shanxi Bethune Hospital, Taiyuan, 030032 Shanxi China; 6 Department of Otolaryngology-Head and Neck Surgery, Shandong Provincial ENT Hospital, Cheeloo College of Medicine, Shandong University, Jinan, 250022 Shandong China; 7 Research Center of Allergy and Immunology, Shenzhen University School of Medicine, Shenzhen, 518055 Guangdong China; 8 Guangdong Provincial Key Laboratory of Regional Immunity and Diseases, Shenzhen, 518055 Guangdong China; 9 Department of Cell biology and Genetics, Basic Medical School of Shanxi Medical University, Taiyuan, 030001 Shanxi China

**Keywords:** Cancer research, CRISPR/Cas9, Gene editing technology, Cancer stem cell, Cancer therapy, Diagnosis of cancer

## Abstract

The 2020 Nobel Prize in Chemistry was awarded to Emmanuelle Charpentier and Jennifer Doudna for the development of the Clustered regularly interspaced short palindromic repeats/CRISPR-associated nuclease9 (CRISPR/Cas9) gene editing technology that provided new tools for precise gene editing. It is possible to target any genomic locus virtually using only a complex nuclease protein with short RNA as a site-specific endonuclease. Since cancer is caused by genomic changes in tumor cells, CRISPR/Cas9 can be used in the field of cancer research to edit genomes for exploration of the mechanisms of tumorigenesis and development. In recent years, the CRISPR/Cas9 system has been increasingly used in cancer research and treatment and remarkable results have been achieved. In this review, we introduced the mechanism and development of the CRISPR/Cas9-based gene editing system. Furthermore, we summarized current applications of this technique for basic research, diagnosis and therapy of cancer. Moreover, the potential applications of CRISPR/Cas9 in new emerging hotspots of oncology research were discussed, and the challenges and future directions were highlighted.

## Background

Cancer is a refractory disease with high mortality and global attention. The malignant tumor causes 1 out of 6 deaths globally thus threatening lives of thousands of human beings [1]. Despite many exciting achievements in the field of cancer therapy, including surgery, radiotherapy, chemotherapy, targeted biotherapy, and new combination therapies, high post-operative recurrence rates and radiation/chemotherapy resistance and harmful toxic side effects continue to be barriers to survival time and quality of life [2]. Studies have shown that cancer is a potentially fatal disease that accumulates multiple genes and alters epigenetics throughout the genome [3]. The mutation of genes in cancer usually drives the proceeding of cancer and impacts the future of tumorigenesis [4]. During the past two decades, a large number of genes related to cancer initiation and progression have been identified by high-throughput sequencing technology [5, 6]. Based on these progressions, gene editing technology holds a big promise for cancer treatment via adjustment of the expression and correction of mutations of genes, which may lead to further breakthroughs in the field of precision oncology.

A number of techniques including zinc finger endonuclease (ZFN) [7], transcription activator-like effector nuclease (TALEN) [8], and the Clustered regularly interspaced short palindromic repeats/CRISPR associated nuclease (CRISPR/Cas) system, are applied to achieve gene editing. Due to its advantages of simple design, rapid implementation, low cost, and strong scalability, researchers consider CRISPR/Cas system as a revolutionary gene editing toolbox that has expanded to almost all genomic targets [9]. Particularly, this system has been widely used in cancer research, and has become a potential approach for cancer diagnosis and treatment [10, 11]. Winning the 2020 Nobel Prize in chemistry is a strong indication of the superiority of CRISPR gene editing.

In this review, we focused on how CRISPR/Cas gene editing technology opens new avenues for cancer basic research, diagnosis, and therapy. We also discussed the current limitations and speculated future directions of the CRISPR/Cas technology in cancer biology.

## Development of the CRISPR/Cas9-based gene editing tools

### Mechanism of the classical CRISPR/Cas9 system

**Fig. 1:**
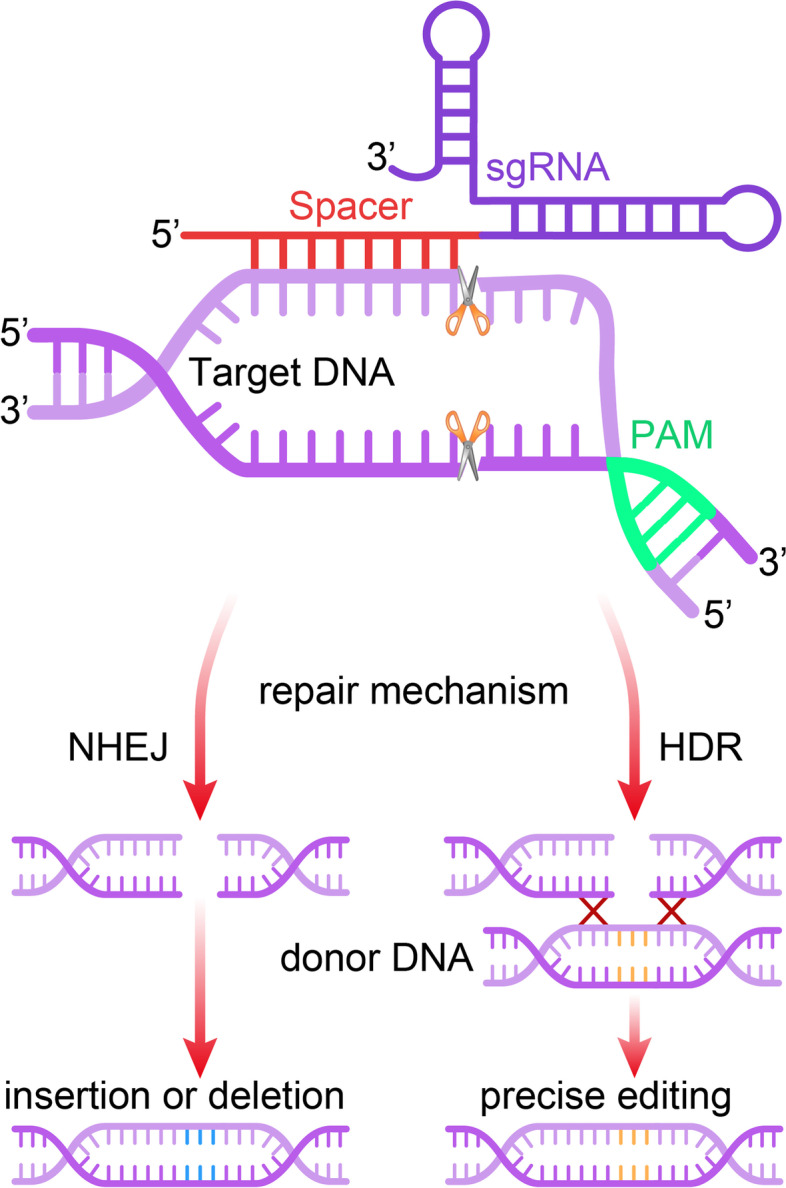
Mechanism of the CRISPR/Cas9 gene editing system. The single guide RNA (sgRNA) directs the Cas9 nuclease to a complementary sequence in the genome where Cas9 will induce a double strand break (DSB). The target genomic locus must be followed by a 5′-NGG-3′motif (protospacer adjacent motif, PAM) for Cas9 to function. DSB are repaired by non-homologous end joining (NHEJ), or by homology directed repair (HDR) in the presence of a DNA repair template, which can be exploited to introduce precise genetic modifications or exogenous sequences

The CRISPR/Cas9 system is a heritable adaptive antiviral immune system of prokaryotes that targets infectious invading viruses and bacteriophages and uses RNA-guided nucleases to cut foreign genetic components [12, 13]. It contains two compartments, one for Cas9 endonuclease and one for single-stranded guide RNA (sgRNA) [14]. The sgRNA directs the Cas9 endonuclease to cleave both DNA strands of the target gene in a sequence-specific manner. DNA cleavage occurs at a sequence 3 base pairs upstream of an “NGG” protospacer adjacent motif (PAM). The genome DNA is repaired by double-strand break (DNA-DSB) repair mechanisms after the cleavage [15]. Therefore, the utilization of the CRISPR/Cas9 gene editing system achieves genome modifications by the introduction of small insertions or deletions (indels) through the relatively error-prone non-homologous end-joining (NHEJ) or the high-fidelity homology-directed repair (HDR) [16] (Fig. 1).

### Development of the CRISPR/Cas9 system and related gene editing tools

**Fig. 2:**
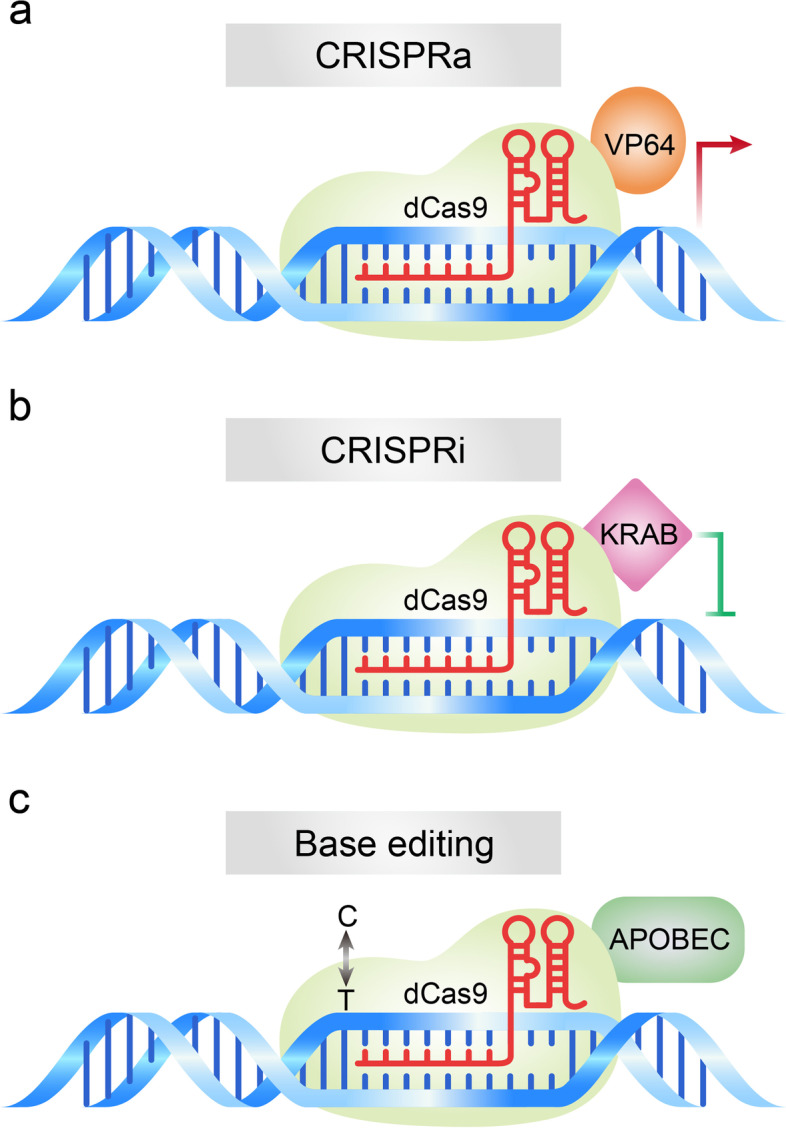
Schematic diagram of dCas9-based gene editing tools. **a** CRISPRa: Fusion of deactivated Cas9 (dCas9) with activation domain VP64 induces the expression of gene of interest. **b** CRISPRi: Fusion of dCas9 with repressor domain KRAB leads to inhibition of gene of interest. **c** Base editing: Fusion of dCas9 with adenosine deaminase or cytidine deaminase enable introduction of point mutation in the genome

In 1987, Japanese scientists discovered some unknown tandem repeats in the *Escherichia coli* genome but did not explore their biological significance [17]. In 2002, these sequences were named as Clustered Regularly Interspaced Short Palindromic Repeats (CRISPR), but the significance of these sequences remained unknown [18]. In 2005, three research teams independently discovered the role of CRISPR loci in adaptive immunity [19–21], then speculated that protospacer-adjacent motifs (PAMs) might direct the type II Cas9 nuclease to cleave DNA [21]. In 2007, Barrangou et al. proved that the CRISPR system is indeed an adaptive immune system and found that phage gene sequences incorporated by bacteria could change the bacteria’s resistance to phages [22]. In 2008, Brouns et al. found that non-coding RNA transcribed from the CRISPR proto-interregional sequence could guide Cas protein to the target-specific part of DNA to play a defensive role [23]. In 2011, Deltcheva et al. revealed that trans-coding crRNA (tracrRNA) was involved in the pre-crRNA processing and maturation process and their study revealed new pathways for crRNA maturation [24]. In 2012, in vitro experiments demonstrated that mature crRNA formed a special double-stranded RNA structure with tracrRNA through base complementary pairing, thus directing Cas9 protein to cause double-stranded fracture on the target DNA [15]. In 2013, Cong and Mali et al. applied the type II Cas system to the cutting of DNA in mammalian cells [14, 25], which paved the way for the application of the CRISPR/Cas9 system in genome modification. In the same year, the Cas9 protein mutant dCas9 (endonuclease-deficient Cas9), which had lost nuclease activity, was first developed [26]. Subsequently, the CRISPR activation (CRISPRa) [27] (Fig. 2a) and interference (CRISPRi) [28] (Fig. 2b) tools were developed by fusing the dCas9 protein with transcription regulators that activate or inhibit gene transcription.

**Fig. 3:**
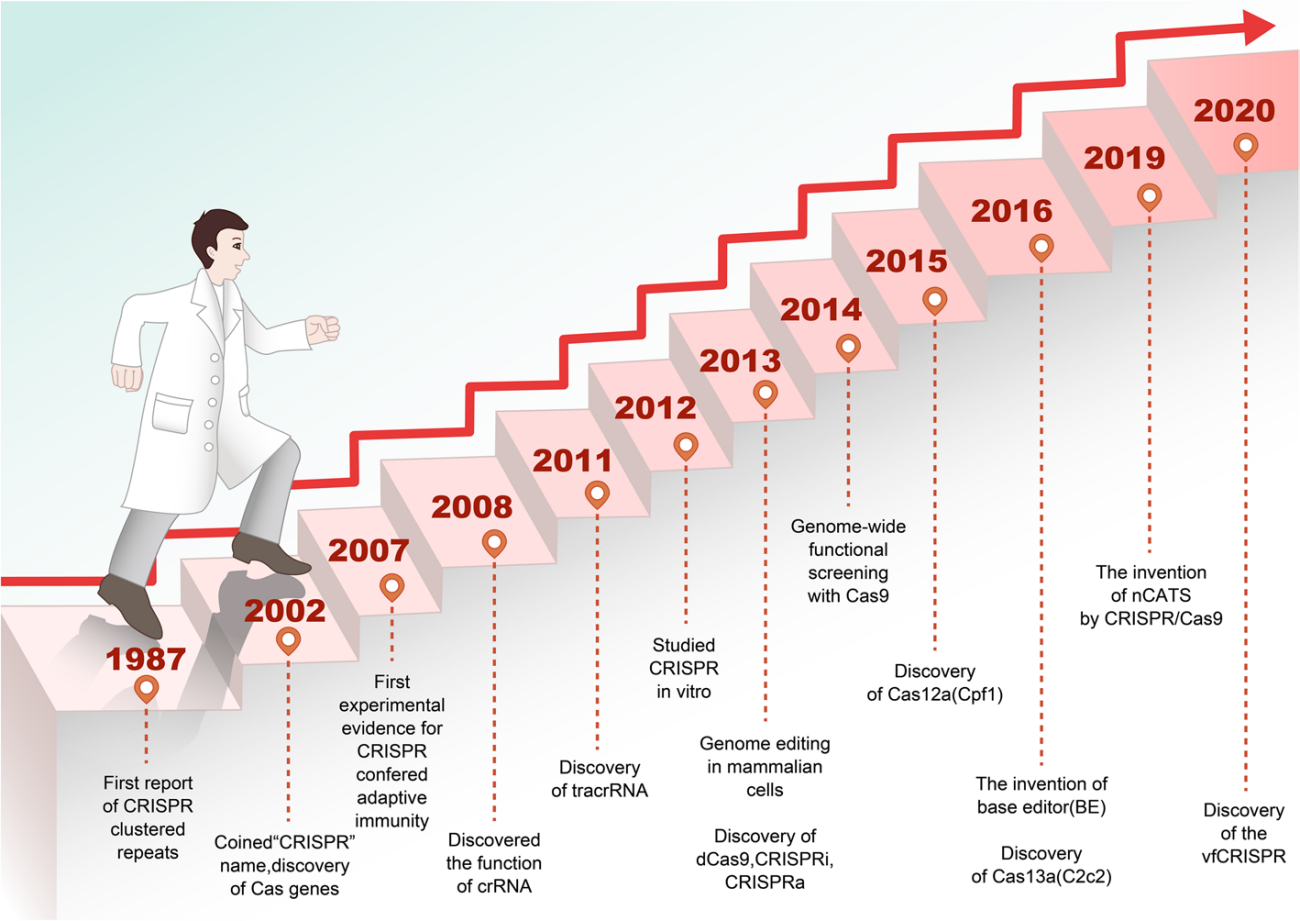
Development history of the CRISPR/Cas9-based gene editing tools. The “CRISPR” repeat sequence was reported in 1987 and named in 2002. In 2012, in vitro experiments demonstrated that mature crRNA formed a special double-stranded RNA structure with tracrRNA by base complementary pairing, thus directing Cas9 protein to cause double-stranded fracture on the target DNA. In 2013, the type II Cas system was applied to the cutting of DNA in mammalian cells, which paved the way for the application of the CRISPR/Cas9 system for gene editing. Since then, the CRISPR/Cas9 technology developed rapidly, and several CRISPR/Cas9-based tools were generated for gene editing at both DNA and RNA levels by 2020

To overcome the problem of unexpected disruptions observed in the CRISPR/Cas9 gene editing system, Komor et al. fused APOBEC (cytosine deaminase) with CRISPR/Cas9. This modified Cas9 makes the C → T (or G → A) conversion under the guidance of gRNA without causing DSB. This base editor could effectively correct a variety of point mutations in the genome [29] (Fig. 2c). Furthermore, the adenine bases Editor (ABE) that converts A - T Base pairs to G - C Base pairs was developed [30]. The team further improved the single-base editing and the CRISPR/Cas9 systems, thus reducing greatly the miss rate of the single-base editor and improving the target range of spCas9 [31, 32]. Interestingly, Gilpatrick et al. used Cas9 and the adapter ligation to develop a nanopore Cas9-targeted sequence (nCATS) for third-generation nanopore sequencing through modifying the target DNA region and change the structure of the target genome, which can read long fragments at low cost [33]. Recently, a caged RNA strategy was developed that allows Cas9 to bind to DNA but does not cleave before light-induced activation. This method was named very fast CRISPR (vfCRISPR) and it creates double-strand breaks (DSBs) at the submicrometer and second scales. The vfCRISPR is very accurate and can edit only one allele at a time, providing a basis for investigating complex genetic traits [34] (Fig. 3). Collectively, these improvements allowed for the transformation of the CRISPR/Cas9 editing tool from a blunt instrument to a precision instrument.

### CRISPR/Cas9 variations

*Streptococcus pyogenes* Cas9 (SpCas9) was the first to be used outside prokaryotic cells [15] and reprogrammed for genome editing in mammalian cells [14]. SpCas9 targeting of DNA relies on the 20-nucleotide-long spacer and on the PAM 5′-NGG (N represents any nucleotide) [35]. In 2015, Kleinstiver et al. obtained the SpCas9-VRER variant with NGA and the SpCas9-VRER variant with NGCG recognition through the error-prone PCR strategy [36]. In the same year, Nishimasu et al. identified the gene editing role of SaCas9 in mammalian cells; it recognizes the PAM of NNGRRT (N refers to A, T, C, G; R refers to A and G) and has excellent cutting activity and targeting accuracy [37, 38]. In 2018, Hu et al. built xCas9 3.7 variants that could identify NGG, NG, GAA, and GAT [39]. Furthermore, a more active SpCas9-NG variant was constructed in the Nureki laboratory, and the identified PAM sequence was extended to NG [40]. Recently, Walton et al. developed a SpCas9 variant SpRY that was almost completely liberated from the constraints of the sequence of PAM [41] (Table 1). These advancements expanded the application scope and accuracy of the CRISPR/Cas9 system in genome editing.

**Table 1:** Comparison of Cas9 variants

Year	Variant of Cas9	PAM(5’to3’)	Application	References
2012	SpCas9	NGG	Multiplex genome editing in mammalian cells.	[14]
2015	SaCas9	NNGRRT	Highly efficient genome-editing by the AAV-SaCas9-gRNA vector system.	[42]
2015	SpCas9-VRER	NGCG	Modification of previously inaccessible sites in zebrafish embryos and human cells	[36]
2017	CjCas9	NNNVRYM	In Vivo Genome Editing with CjCas9 in TA Muscles of Dmd Knockout Mice	[43]
2018	xCas9–3.7	NG, GAA, GAT	Base substitution of C•G → T•A and A•T → G•C for the pathogenic mutation sites	[39]
2018	SpCas9-NG	NG	Efficient and accurate genome editing in mouse zygotes and somatic culture cells	[44]
2018	evoCas9	NGG	Limits the unspecific cleavage of a difficult-to-discriminate off-target site and fully abrogated the cleavage of two additional off-targets	[45]
2020	SpRY	PAM sequence has not been characterized	Precise editing to extend almost to the entire genome	[32]

**Fig. 4:**
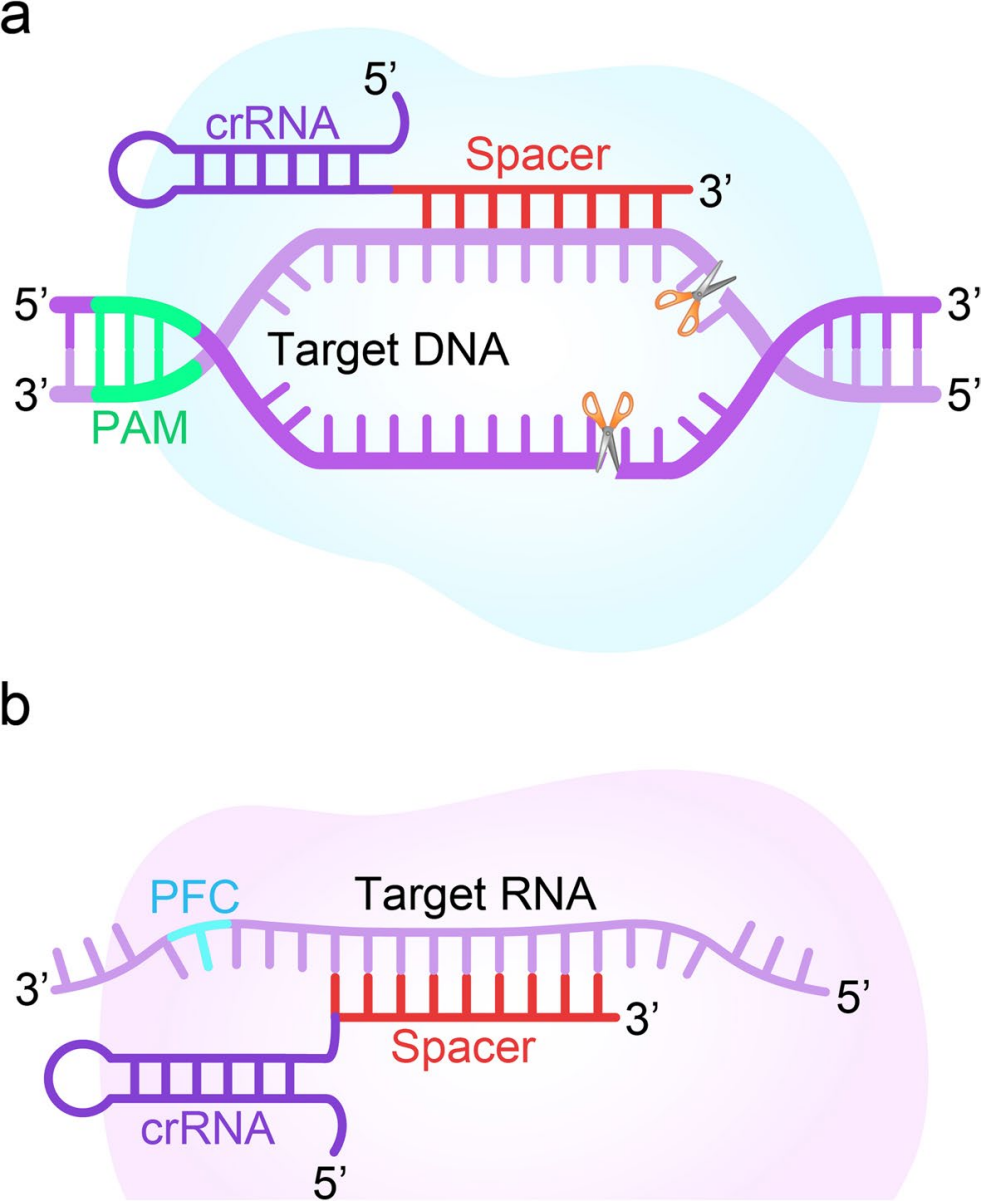
Schematic diagram of CRISPR/Cas12 and CRISPR/Cas13 systems. **a** CRISPR/Cas12: the crRNA directs the Cas12 nuclease to a complementary sequence in the genome where Cas12 will induce a double-strand break (DSB) of DNA. The target genomic locus must be followed by PAM for Cas12. **b** CRISPR/Cas13: the crRNA directs the Cas13 nuclease to a complementary sequence in the genome where Cas13 will induce a single-strand break of RNA. The target sequence must be followed by a protospacer flanking sequence (PFS) for Cas13

Like CRISPR/Cas9, Cas12a (also known as Cpf1) has been applied to genome editing for its ability to generate targeted DSB [46] (Fig. 4a). However, Cas12a requires only crRNA guidance for DNA targeting, and its enzyme recognizes a T-rich PAM upstream of the target region and cleaves DNA at the PAM-distal site [47]. Chen et al. combined isothermal amplification of recombinase polymerase with LbCas12a to create a method called DNA Endonuclease Targeted CRISPR Trans Reporter (DETECTR), which achieves attomolar sensitivity for nucleic acid detection. They proved that DETECTR could rapidly and specifically detect HPV in human patient samples, providing a more efficient platform for nucleic acid-based diseases [46]. Therefore, Cas12a offers a novel approach to genome editing with its unique cutting mechanism that enhances and extends the CRISPR toolkit.

The type-V CRISPR effector Cas12b (formerly known as C2c1), is a dual-RNA-guided nuclease containing a single RuvC domain and requiring both crRNA and tracrRNA [48]. Cas12b generates staggered ends distal to the protospacer adjacent motif site in vitro when reconstituted with the crRNA/tracrRNA duplex [49]. Importantly, Cas12b offers a smaller size than the most widely-used SpCas9 and Cas12a, making it suitable for in vivo delivery of adeno-associated virus (AAV)-mediated gene therapy [47, 50]. Moreover, Cas12b recognizes simpler PAM sequences than the small-sized Cas9, such as SaCas9 and CjCas9 [48], which can markedly expand the targeting range of the genome. Teng et al. reported that AaCas12b can be used for mammalian genome engineering, enabling a variety of functional applications such as single and multiple genome-editing, gene activation, and generation of mutant mouse models [51]. Recently, a CRISPR/Cas12-based diagnostic tool for the rapid and visual detection of SARS-CoV-2 from extracted RNA from the patient sample has been reported [52]. These studies revealed the functional diversity achieved in different pathways of CRISPR/Cas evolution that further extends the application of the CRISPR/Cas9.

The class II type-VI CRISPR/Cas13 system was found to be useful for RNA editing of eukaryotic cells [53, 54]. Cas13(C2c2) is guided by a single CRISPR RNA and can be programmed to cleave single-stranded RNA targets carrying complementary protospacer [55] (Fig. 4b). Furthermore, the RNA Editing for Programmable Adenosine to Inosine Replacement (REPAIR) system has been developed through the improvement of the Cas13 effector and can edit full-length transcripts containing pathogenic mutations [56]. Functionally, the CRISPR effector Cas13 has effective antiviral ability for single-stranded RNA (ssRNA) viruses [57]. Besides, Cas13 is used as a tool to target and knockdown viruses and to investigate their replication, localization, and evolution. Particularly, the catalytic death mutant of Cas13 (dCas13) can be used to study viral RNA localization, and dCas13 fusion proteins with RNA editing capabilities used to functionally characterize specific viral polymorphisms [48, 58]. These studies provided tools to visualize and interfere with virus replication with high precision.

In 2017, Gootenberg et al. developed a viral detection technology based on the CRISPR/Cas13 system that could make the cut RNA bands form visual clues and display them visually. They named it Specific High Sensitivity Enzymatic Reporter Unlocking (SHERLOCK) [59]. SHERLOCK has since been applied in detecting RNA virus outbreaks such as Lassa fever, Ebola, Zika, and Dengue. It is cheaper and faster with improved efficiency of virus detection, hence helping to reduce the lethality of viruses. In the recent outbreak of novel coronavirus SARS-COV-2 that posed a major challenge to global health, Zhang Feng’s team used SHERLOCK to design a new virus detection method to maximize the specificity and accuracy of detection. Moreover, Freije et al. combined the antiviral activity of Cas13 with its diagnostic capability to build a powerful and rapidly programmable diagnostic and antiviral system, named CARVER (cas13-assisted restriction of virus expression and readout), to detect and eliminate RNA viruses in human cells [57]. Notably, researchers found that the CRISPR/Cas13d gene editing system delivered by adeno-associated virus AAV could edit and clear the new coronavirus, and this method could effectively deal with the possible mutation of the virus [60]. Taken together, Cas13 may be a potential tool for clinical diagnosis and treatment of diseases caused by viral infections.

## Application of the CRISPR/Cas system in cancer research

Cancer initiation and progression involved in mutations and dysregulated expression of a series of genes [61], including oncogenes, tumor suppressor-genes, chemoresistant genes, metabolism-related genes, and cancer stem cell-related genes. The ultimate goal of cancer treatment is to restrain tumor growth and progression by specifically correcting mutations and restoring expression of dysregulated genes [62]. The CRISPR/Cas9 gene editing system has been widely used in the basic research of cancer, and some encouraging progress has been achieved.

### DNA-based knockout/in

#### Oncogenes

**Fig. 5:**
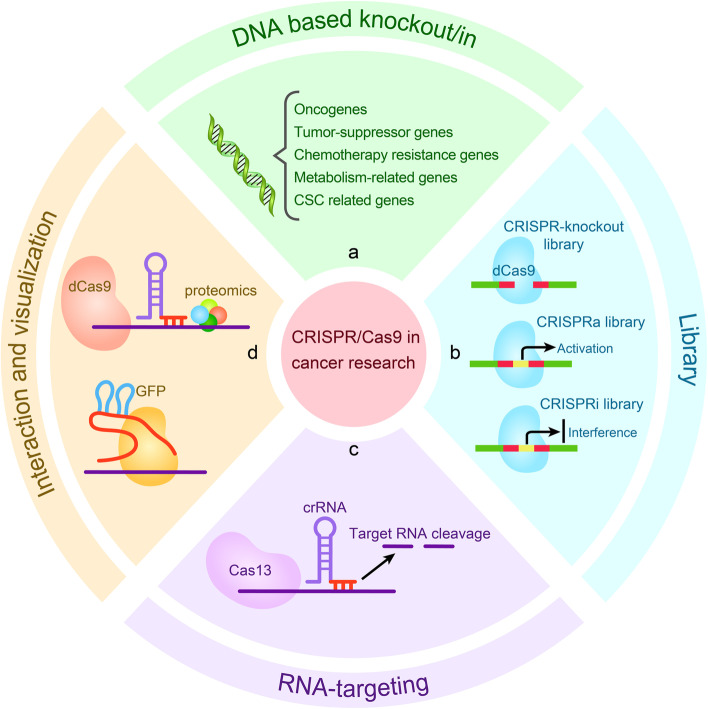
Applications of CRISPR/Cas9-based gene editing tools for basic research of cancer. **a** CRISPR/Cas9-mediated knockout or knockin was applied to identify and verify of functional gene, including oncogene, tumor-suppressor gene, chemoresistant gene, metabolism-related gene, and cancer stem cell-related gene. **b** CRISPR/Cas9 library screen for drug target and functional gene. **c** Application of the CRISPR/Cas13 system for RNA targeting. **d** In vivo biotinylated dCas9 protein was used for interaction research, and dCas9 protein fused with fluorescent markers such as GFP was used for imaging analysis

Oncogenes are regulated differently from normal genes and can promote malignant transformation of normal cells [63]. The CRISPR/Cas9 system provides a valid measure for deletion, interfering with the expression, and changing the activity of oncogenes, thereby inhibiting tumor growth [64]. The CRISPR/Cas9-mediated knockout of CD133 downregulated vimentin expression in colon cancer cells, significantly reduced cell proliferation and colony formation, and showed significant inhibitory effects on cell migration and invasion [65]. Knockdown of miR-3064 using CRISPR/Cas9 technology significantly inhibited the proliferation, invasion, and tumorigenic capacity of pancreatic cancer (PC) cells [66]. Besides, using CRISPR/Cas9 knockdown of oncogenic mutant EGFR alleles, the growth and proliferation of lung cancer cell lines H1975, A549, and H1650 were found to be inhibited, and the tumor size of xenografted mice implanted with H1975 or A549 cells was reduced [67]. Knockdown of the FAK gene in NSCLC cells with KRAS mutations using CRISPR/Cas9 resulted in detectable DNA damage and increased sensitivity to radiotherapy [68]. Furthermore, the CRISPR/Cas9-mediated deletion of the E3 ubiquitin ligase UBR5 in an experimental murine model of triple-negative breast cancers (TNBC) remarkably inhibited tumor growth and metastasis in vivo [69] (Fig. 5a). These studies indicated that the CRISPR/Cas9 gene editing system is an effective tool for identifying oncogene and evaluating the therapeutic potential of oncogene targeting.

#### Tumor-suppressor genes

Inactivation of tumor suppressor genes is a significant feature in the initiation and progression of cancer [70]. The silencing, deficiency, or mutation of tumor-suppressor gene activates oncogenes, leading to tumor initiation and progression [71]. Notably, the application of the CRISPR/Cas9 system has reformed a revolution in cancer research by enabling the rapid validation of tumor-suppressor gene in vitro and in vivo.

The mutation of NF2 (Neurofibromin 2), a gene to inhibit tumorigenesis, was found in malignant pleural mesothelioma (MPM). Compared to the untreated cells, an NF2-knockout human mesothelial cell line, MeT-5A (NF2-KO) showed enhanced migration and invasion abilities [72]. Similar phenomenon was observed on MFN2 (mitochondrial GTPase mitofusin-2) knockout cells in lung cancer via CRISPR/Cas9 [73]. Deletion of multiple tumor suppressor genes (including p53, Nf1, Pten, and Ptch1) in the mouse brain by CRISPR/cas9 resulted in the development of glioblastoma [11]. The loss of LATS1/2 in many mouse cancer cell lines through CRISPR/Cas9 significantly increased anchorage-independent growth in pancreatic cancer Panc02, prostate cancer MyC-Cap, breast cancer 168FARN and 67NR cells, and growth of colon cancer CT26, glioma GL261, and bladder cancer MB49 [74] (Fig. 5a). These studies showed that the use of CRISPR/Cas9 technology is able to identify tumor-suppressor gene.

Furthermore, CRISPR/Cas9 technology has shown cancer therapeutic benefit by repairing inactivated tumor-suppressor genes. Moses et al. used the CRISPR/dCas9 in combination with the trans-activator VP64-p65-Rta (VPR) to activate PTEN expression in cancer cells with low-level PTEN expression. Results showed that the PTEN expression level was increased in melanoma and TNBC cell lines by the dCas9-VPR system, and the activation of PTEN obviously inhibited downstream oncogenic pathways [75]. Artegiani et al. found that after loss-of-function of BAP1 by CRISPR/Cas9 in normal human cholangiocyte organoids, the cells became more motile and fused with other organoids, features that resemble the metastatic invasion of cancer. Interestingly, they restored the catalytic activity of BAP1 in the nucleus and rescue the cellular and molecular alterations [76].

#### Chemotherapy resistance genes

The development of resistance of cancer cells to chemotherapeutic drugs is a major obstacle to cancer treatment. The main mechanism of chemoresistance is dysregulation of chemoresistance related genes [77]. Therefore, identifying chemoresistance related genes and modulating their expression levels or functions are key to the elimination of chemoresistant cancer cells.

CRISPR/Cas9-mediated knockdown of NRF2 in a lung cancer cell xenograft mouse model had enhanced sensitivity to cisplatin and carboplatin [78]. Furthermore, Gao et al. found that CRISPR/Cas9-mediated knockdown of SKA3 enhanced the sensitivity of laryngeal cancer cells to cisplatin in preclinical model [79]. Knockdown of RSF1 using CRISPR/Cas9 technology significantly increased the sensitivity of H460 cells to paclitaxel [80]. In contrast, knockdown of ERCC1 decreased the sensitivity of lung cancer cell lines to cisplatin [81]. The knockdown of aurora B (AURKB) in NSCLC cell lines by CRISPR/Cas9 also restored the expression of the tumor suppressor gene TP53 and sensitivity to cisplatin and paclitaxel [82]. Therefore, the use of CRISPR/Cas9 technology enable to identify and validate chemoresistant genes, which are of great significance in the clinical treatment of cancer.

#### Metabolism-related genes

Tumor cells are dependent on an adequate supply of energy to support proliferation, migration, and invasion as occurs with metastatic cells. The research suggested that metabolic reprogramming, the regulation of energy metabolism to promote rapid cell growth and proliferation, is the new Hallmark of cancer [63]. Cancer cells tend to favor the “Warburg effect”, which promotes glycolysis or aerobic glycolysis, even in the adequate supply of oxygen [83]. In addition to glucose metabolism disorders, abnormal lipid metabolism, amino acid metabolism, mitochondrial biogenesis, and other bioenergy metabolism pathways also exist in cancer cells [84]. Therefore, understanding the energy metabolism mechanism might provide us with new ideas to target energy production pathways in cancer treatment.

Common markers expressed in high levels in cellular hypoxia, glucose transporter-1 (GLUT-1) and hypoxia-inducible factor-1α (HIF-1α), have been associated with the biological behavior of cancer [85, 86]. Under the hypoxic stress condition, these two proteins are important for glucose uptake and glycolysis in laryngeal cancer cells [87]. Lu et al. conducted HIF-1α and GLUT − 1 gene knockout in HEp-2 cells by CRISPR/Cas 9 system, leading to decreased proliferation, migration, and invasion. They found that HIF-1α and GLUT-1 gene knockout resulted in a significant reduction in glucose uptake and lactic acid of HEp-2 cells [88].

Using an impartial genome-wide CRISPR/Cas9 screen, Gallipoli et al. revealed that glutaminase (GLS) existing in glutamine metabolism as a primary enzyme had a synthetically lethal effect with FLT3 tyrosine kinase inhibitors (TKI) treatment. Subsequently, combined with complementary metabolomic and gene-expression analysis, they indicated that there is a metabolic dependency relationship between glutamine metabolism and FLT3 internal tandem duplication (FLT3ITD) cells in acute myeloid leukemia (AML) after FLT3 TK inhibition. They used these approaches to explore AML subtypes driven by other tyrosine kinases (TK) activating mutations and verified the possibility of GLS as a clinically therapeutic target in AML [89].

The chemotherapeutic drug methotrexate is cytotoxic through inhibition of the synthesis of nucleotides which inhibit the DHFR enzyme (dihydrofolate reductase) that produces tetrahydrofolate (THF) and reduces its potential efficiency thereby leading to cell death. Kanarek et al. conducted a CRISPR/Cas9-based screening that generated FTCD, an encoded enzyme that is essential for histidine catabolism (formimidoyltransferase cyclodeaminase). They found that the absence of multiple genes in the histidine catabolism pathway significantly reduced sensitivity to methotrexate in cultured cancer cells. Thus, the flow through the histidine degradation pathway could be increased by dietary supplementation of histidine in vivo, which enhances the sensitivity of leukemic xenografts to methotrexate [90] (Fig. 5a). This application of CRISPR/Cas technology in tumor metabolism has brought new insights into the treatment of cancer.

#### Cancer stem cell-related genes

Cancer stem cells (CSC) are self-renewing cells in tumors that can produce heterogeneous tumor cells, which play critical roles in cancer initiation, progression, recurrence, and therapeutic resistance [91]. Since CSC may be derived from oncogene reprogramming and the dynamic characteristics of cancer cells, the identification of CSC-related gene is speculated to generate new cancer treatment targets [92]. Nowadays, the application of CRISPR technology in tumor stem cells has provided a new direction for clinical tumor treatment.

Ovarian cancer stem cells (OCSC) lead to a poor prognosis of ovarian cancer. Nanog has been identified as the key gene that maintains CSC pluripotency and self-renewal capability [93]. Androgen receptors (AR) are involved in the malignant behavior of other tumors [94]. Ling et al. constructed a green fluorescent protein (GFP) labeled Nanog cell model in ovarian cell lines (A2780 and SKOV3) using the CRISPR/Cas9 system, they found that the interaction of Nanog with the AR signaling axis may induce or contribute to the regulation of OCSC [95]. In another study, Yang et al. used CRISPR/Cas9 technology to knockdown the transcription factor YB-1 gene in cancer stem cells. They observed that the absence of YB-1 inhibited the proliferation of breast cancer and melanoma stem cells, leading to cell cycle arrest, apoptosis, and irreversible differentiation [96]. In colorectal cancer, aberrant Wnt signaling is critical for the development and maintenance of cancer stem cells [97]. Zhan et al. found that APC truncation mutations generated by CRISPR/Cas9 and MEK inhibitors synergistically enhanced Wnt responses in a CRC model. Using CRC-like organs derived from patients, they demonstrated that MEK inhibition leads to an increased Wnt activity and enhanced genetic markers associated with stemness and cancer recurrence [98]. This provides a potential solution for the treatment of colorectal cancer. Hwang et al. used CRISPR/Cas9 to knockdown REG4 in colorectal cancer spheres containing both APC and KRAS mutations, and results showed that knockdown of REG4 inhibited Wnt/β-linked protein signaling and thus effectively suppressed CSCs properties [99].(Fig. 5a). These findings explored the regulatory mechanisms of cancer stem cell stemness from multiple perspectives and provide new ideas for the CSC targeting treatment of cancer.

### Using CRISPR/Cas9 library for screening functional genes in cancer cells

Cancer cell genomes carry a diversity of genetic aberrations that accumulate from congenital and acquired mutations and are triggered by successive clonal expansions [63]. Identifying the genes that drive tumor evolution can clarify the initiation and development of cancer [3]. Large-scale genomic screening is a powerful tool to detect the mutated genes that cause various cancers [63]. Using CRISPR to perform functional genomic screening can reveal phenotype changes after drug treatment or other stimulation, thereby identifying new target for cancer treatment [100].

Large-scale screening using CRISPR/Cas9 knockout libraries is widely used in gene loss-of-function studies. In 2014, Shalem et al. constructed a sgRNA library targeting 18,080 genes in the human genome and named it Genome-wide CRISPR/Cas9 knockout Library (GecKO). Using this system, they screened candidate genes that respond to vemurafenib (a therapeutic RAF inhibitor) in the human melanoma cell line A375, and six candidate genes were identified, including NF1, MED12, NF2, Cul3, TADA2B, and TADA1 [101]. In 2015, Chen et al. performed a genome-wide CRISPR/Cas9-mediated loss-of-function screen in a mouse model of tumor evolution. They used a lentiviral mouse sgRNA library named mGeCKOa, which containing 67,405 sgRNAs targeting 20,611 protein-coding genes and1175 microRNA precursors. A total of 5 protein-coding genes and 2 microRNAs were identified as metastasis-suppressive genes, including Nf2, Pten, Cdkn2a, Trim72, Fga, miR345, and miR-152 [102].

The tumor suppressor gene ATRX is frequently mutated in a variety of tumors, including hepatocellular carcinoma (HCC) and glioma [103, 104], and has a minimal response to current therapies. Liang et al. revealed that the checkpoint kinase WEE1 was a potential therapeutic target for ATRX mutant cancers using CRISPR/Cas9 whole-genome screening technology. Subsequent experiments revealed that treatment with the WEE1 inhibitor AZD1775 robustly inhibited the growth of several ATRX-deficient HCC cell lines in vitro, as well as in vivo xenografting. Thus, the discovery of a synthetic lethal relationship between WEE1 and ATRX could be widely used for therapeutic applications in human tumors [105]. Additionally, researchers used the CRISPR/Cas9 library screening technique to knockout more than 3000 genes involved in T-cell metabolism. They tested the function of these genes in a mouse anti-tumor ACT (Adoptive cell therapy) model and found that damage to the gene encoding the REGNASE-1 enzyme caused more T-cells to infiltrate tumor tissue. Subsequently, they used CRISPR/Cas9 knockout library to destroy about 20,000 genes in REGNASE-1-deficient CD8+ T cells. Their study indicated that the loss of REGNASE-1 protein prolongs the survival of anti-tumor CD8+ T cells, enhances their function, and enable T cells to fight cancer better and more effectively [106]. Moreover, Zhu et al. developed a CRISPR/Cas9 strategy using paired gRNAs (pgRNAs) for large segment deletions to identify functional long non-coding RNAs in cancer cells. By applying this method, the researchers identified 51 lncRNAs that positively or negatively regulate the growth of human cancer cells [107]. Subsequently, the same team developed an alternative loss-of-function screen for 10,996 multiple exon lncRNA splice sites in chronic myeloid leukemia cells K562 and identified 230 lncRNAs associated with cell survival or proliferation [108] (Fig. 5b).

CRISPRi (CRISPR inhibition) is another important CRISPR/Cas9-based technology that was developed for loss-of-function screening in cancer research. Since CRISPRi functions only in a small range (1 kb) around the target transcription start site (TSS) [109], and dCas9 blocks only 23 bp of the targeted sequence [37], CRISPRi can interfere precisely with any lncRNA gene. Liu et al. developed a CRISPRi library targeting 16,401 lncRNA loci to screen cell lines, including iPSCs (human induced pluripotent stem cells), and transformed cell lines. They identified 499 loci that are required for robust cell growth [110]. Jost et al. used a CRISPRi/a-mediated chemogenetic screen to identify a target of anticancer drug Rigosertib. Their results indicated that tubulin with structure-directed mutations at the interface with rigosertib developed resistance to rigosertib, and it was determined that rigosertib kills cancer cells by destabilizing microtubules [111]. This work confirmed the importance of CRISPR-based chemical gene screening in identifying physiologically relevant targets for drugs. Raffeiner et al. used a pooled library screen of dCas9 fused to the efficient transcriptional repression domain of the MXD1 protein to identify non-coding sites required for the growth of the human lymphoblastoid cell lines P493–6 and RAMOS. The study also provides additional CRISPRi-based tools to facilitate genetic perturbation of noncoding targets [112] (Fig. 5b). In summary, these approaches open the door to both coding and non-coding RNA screening.

In contrast to pooled libraries for knockdown screening, sgRNAs in CRISPR activation (CRISPRa) libraries target the promoter sites of target genes. CRISPRa in particular has enormous potential for the elucidation of drug resistance mechanisms in cancer cells, which are thought to arise frequently from gain-of-function events. The use of CRISPRa screening in BRAF (V600E) melanoma cells for resistance to the BRAF inhibitor PLX-4720 not only reproduced previously known resistance mechanisms, such as EGFR and ERK pathway activation but also revealed novel resistance mechanisms regarding G protein-coupled receptors [113]. Furthermore, Melanoma was screened for CRISPRa library and positively selected by the BRAF protein kinase inhibitor vemurafenib (PLX). EGFR, PCDH7, ITGB5, ARHGFE1, BCAR3, GPR35, and TFAP2C were identified as PLX-resistant genes. Activation of these genes may be related to the ERK pathway, leading to PLX resistance [114]. Moreover, researchers constructed a genome-wide CRISPRa library targeting 14,701 lncRNA genes. By screening with this library, lncRNA GAS6-AS2 was found to lead to Ara-C resistance in multiple cancers, including AML [100]. In a recent study, Wang et al. developed new cancer immunotherapy, MAEGI (Multiple Activation of Endogenous Genes as Immunotherapy), which uses CRISPR activation technology to directly activate endogenous mutant genes, amplify specific signals from cancer cells, and induce effective adaptive anti-tumor immunity. This is a versatile and highly scalable strategy that is effective against multiple cancer types, including those currently resistant to immunotherapy [115] (Fig. 5b). Collectively, CRISPRa library screening provides more advanced strategies for tumor research and is a good guide for the clinical treatment of cancer.

### Application of CRISPR/Cas13 for RNA targeting in cancer

With the development of gene editing technology, researchers demonstrated that the class 2 type VI RNA-guided RNA-targeting CRISPR/Cas effector Cas13 (previously known as C2c2) can be engineered for mammalian cell RNA knockdown [58] (Fig. 5c). This technology is also increasingly being used in cancer research. Qi et al. built a light sensor that effectively induced Cas13a protein expression after blue light irradiation. They select the lncRNA Metastasis-associated Lung Adenocarcinoma Transcript 1 (MALAT1) as the functional target. Their results showed that the expression of MALAT1 was significantly downregulated by the light-switchable CRISPR/Cas13a system in bladder cancer cells [116]. Recently, Wang et al. overexpressed the LwCRISPR/Cas13a by lentivirus in glioma cells reveals that crRNA-EGFP induces a “collateral effect” after knocking down the target gene in EGFP-expressing cells. This study expands the application scope of the CRISPR/Cas13a system [117]. Taken together, these studies demonstrated that the CRISPR/Cas13a system provides a new approach for RNA manipulation in cancer cells.

### Using CRISPR/dCas9 for interactions and visualization research

**Fig. 6:**
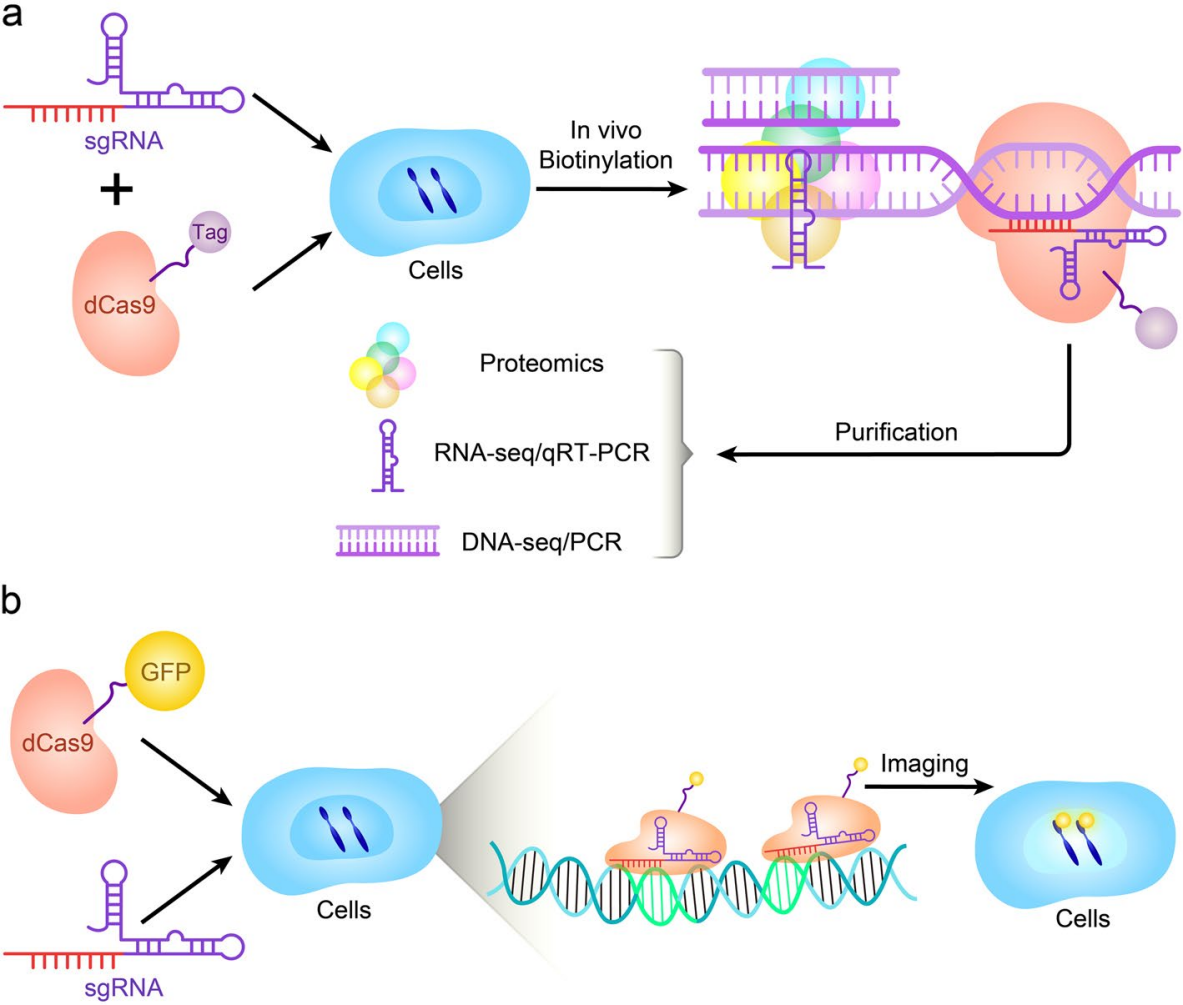
Schematic diagram of dCas9-based methods for molecular interactions and visualization research. **a** dCas9 mediated capture of chromatin complex and downstream analysis. **b** dCas9-mediated imaging of genomic elements in living cells

Importantly, identifying molecules associated with genomic regions of interest in vivo helps to understand locus function (Fig. 5d). Researchers have established an enChIP (engineered DNA-binding molecule-mediated ChIP) system that uses catalytically inactive dCas9 to purify genomic sequences designated by specific gRNAs [118]. For example, enChIP was used for biochemical analysis of epigenetic regulation and transcription at specific genomic loci in living cell lines including HT1080 (a human fibrosarcoma cell line) and K562 (a human leukemia cell line) [119]. Recently, a CRISPR affinity purification in situ of regulatory elements (CAPTURE) system has been developed to identify locus-specific chromatin-regulating protein complexes and long-range DNA interactions. The CAPTURE system can isolate chromatin interactions at a single-copy genomic locus using an in vivo biotinylated dCas9 protein and sequence-specific guide RNAs [120] (Fig. 6a). The ability of CAPTURE to allow the isolation and analysis of the factors that regulate DNA offers multiple possibilities for studying how different proteins control genomic function in cancer cells and stem cells. It also provides an entirely new avenue for discovering new drug targets.

Visualization of chromosome dynamics and shapes in a live cell is very important in the field of cell biology. The copy number of a specific chromosome in cancer cells is usually abnormal, so detecting the chromosome copy number can help cancer diagnosis. In the interphase, each chromosome exists in its nuclear region and can be imaged by fluorescence in situ hybridization (FISH) using sequence-specific probes of different colors [121]. Nonetheless, such chromosome mapping is only applicable to fixed cells and cannot be dynamically monitored in live cells. Recently researchers fused catalytically inactivated Cas9 (dCas9) with fluorescent markers (such as GFP) turning dCas9 into a customizable DNA marker that is compatible with live cell fluorescence microscopy [121] (Fig. 6b). CRISPR-based imaging has many advantages over other imaging technologies because gRNA is easy to design and implement, thereby it can be programmed for different genomic sites and can detect multiple genomic sites at the same time [122]. Alternatively, gRNA and protein-interacting RNA aptamers can be fused, the latter will recruit specific RNA binding proteins (RBP) labeled with fluorescent proteins to visualize target genomic sites [123]. Therefore, the fluorescent CRISPR system has been used for the dynamic tracking of genomic loci and mapping of chromosomes in living cells. In a recent study, Artegiani et al. developed a new tool called CRISPR-hot (CRISPR/Cas9-mediated homology-independent organoid transgenesis) that fluoresces and visualizes specific genes in human organs. They used CRISPR-hot to insert fluorescent tags into the DNA of human-like organs [124]. These new techniques open a wide outlook for studying the real-time dynamic cellular processes in the cell, tissues, and even organs.

## Application of CRISPR/Cas9 in cancer diagnostics

**Fig. 7:**
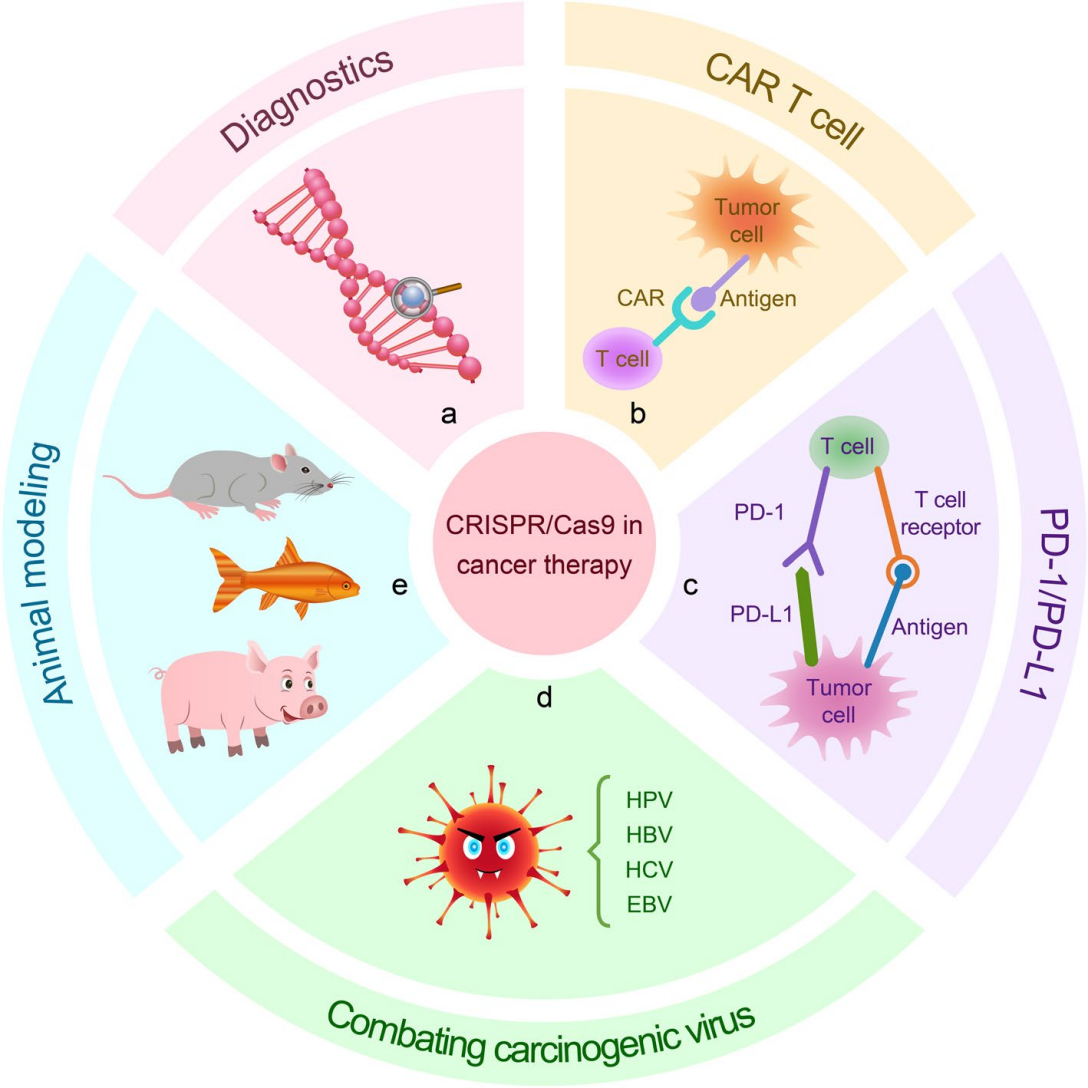
Applications of CRISPR/Cas9 gene editing tools for diagnosis and therapy of cancer. **a** Using CRISPR/Cas9-based diagnostic system SHERLOCK and DETECTR for detecting cancer. **b** CRISPR/Cas9 edits immune cells in vitro, and then these cells were administrated to patients to combat against cancer. **c** Knockout of inhibitory receptors PD-1 by CRISPR/Cas9 technology improves the efficacy of cancer immunotherapy. **d** Viral genome-specific Cas9-sgRNA eliminates oncogenic virus. **e** Establishing in vivo tumor model with multiple gene mutations with CRISPR/Cas9 gene editing tools

Early detection and treatment of cancer can reduce mortality and improve the life quality of patients after treatment. Although several techniques are widely used for cancer detection, they need improvements in terms of sensitivity, specificity, and speed. Therefore, identifying sensitive genes through genetic diagnosis is key to the prevention of cancer [125] (Fig. 7a). To overcome these problems, Gootenberg et al. established a CRISPR-based diagnostic system called SHERLOCK (Specific High Sensitivity Enzymatic Reporter UnLOCKing). This system consists of the RNA-guided RNase Cas13a (induces robust non-specific single-stranded DNA (ssDNA) trans-cleavage as a collateral effect) and a reporter signal (released after RNA cleavage), which showed high sensitivity in mutation detection of BRAF V600E and EGFR L858R in mammalian cells [59]. Another similar system named DETECTR (DNA endonuclease-targeted CRISPR trans reporter) consists of Cas12a and recombinase polymerase amplification (RPA) and is used as a detection tool to screen for viral infections in cancer and to amplify micro-samples. The system is rapid and inexpensive for detecting high-risk HPV types, such as HPV16 and HPV18, in samples infected with many different types of HPV [46, 126].

miRNAs are involved into many diseases and have potential value in the diagnosis and treatment of cancer. However, in view of the complexity, the available miRNAs detection methods have low sensitivity and are of high cost thus, need for improvement. It has been shown that a new isothermal amplification platform based on CRISPR/Cas9 technologies can be used for efficient detection of miRNAs [127]. This was the first time the CRISPR/Cas9 method has been used for miRNA detection. The further development of CRISPR detection technology will provide a rapid, scalable, and high-resolution platform for the diagnosis of cancer.

## Application of CRISPR/Cas9 in cancer therapy

In addition to making rapid progress in basic research of oncology, CRISPR/Cas-mediated genome editing also has broad prospects in cancer treatment. Tumorigenesis is a multistep process involving complex interactions between cancer cells and the host immune system [128]. The combination of the CRISPR/Cas technique with cancer immunotherapy and its application to combating carcinogenic virus infection offers great promise.

### Chimeric antigen receptor T(CART) cells

Cancer immunotherapy is another widely recognized treatment after surgery, chemotherapy, and radiation therapy. Adoptive T cell immunotherapy, especially chimeric antigen receptor (CAR) T cell therapy, has ushered in a new era of cancer therapy, especially after the FDA approved Kymriah and Yescarta (CD19-oriented CAR T cells in B-cell leukemia and lymphoma) [129] (Fig. 7b). Clinical trials have shown that CAR T-cell therapy can alleviate clinical symptoms in patients with a variety of hematologic and solid cancers, particularly in relapsed/refractory acute lymphoblastic leukemia (ALL) and multiple myeloma, providing hope of a cure for patients with relapsed and refractory hematologic cancers [130–132]. Although the widely used autologous CAR T cells have shown promising results in cancer treatment, there are still limitations that affect their therapeutic efficacy. The combination with CRISPR/Cas9 technology would bridge the gap in T-cell engineering.

An MSKCC (Memorial Sloan Kettering Cancer Center) group has used CRISPR/Cas9 technology to construct more effective CAR-T cells using targeted insertion of the CAR gene delivered to specific locations. It is a precise method that kills cancer cells in the long term, is safer, and enhances the effectiveness of T cells [133]. Researchers have developed novel antigen-specific immunotherapies using CAR-T cell-based combined with CRISPR/Cas9 technology. They used the CRISPR/Cas9 tool to remove a specific protein called CD33 from healthy cells. Healthy stem cells that lacked CD33 were able to function normally as well, making CD33 a unique marker for leukemia cells and enabling CAR-T cell therapy to easily identify and attack cancer cells [134].

Researchers at the University of Pennsylvania conducted a human clinical trial in which they used CRISPR technology to delete two genes, TCRα (TRAC) and TCRβ (TRBC), encoding the endogenous T cell receptor (TCR) chain in T cells to reduce TCR mismatch and enhance the expression of a synthetic cancer-specific TCR transgene (NY-ESO − 1). They then deleted a third gene encoding PD-1 (PDCD1) to enhance antitumor immunity. This approach may help avoid host-mediated immunity and thus provide patients with an anti-leukemic effect without the fear of graft-versus-host disease (GVHD). This was the first demonstration of the ability of CRISPR/Cas9 technology to target multiple human genes simultaneously [135]. In a study using CRISPR/Cas9 to disrupt GM-CSF, CART19 cells deficient in GM-CSF were constructed with enhanced cellular function, increased antitumor activity, and improved overall survival. Additionally, after neutralizing GM-CSF using lenzilumab, there was durable control of leukemia and reduced cytokine release syndrome (CRS) and neuroinflammation in patient-derived xenografts. They plan to conduct a phase II clinical study using a combination of lenzilumab and CART19 cell therapy [136]. These studies confirm the feasibility of CRISPR/Cas9 gene editing in cancer immunotherapy.

### PD-1/PD-L1 targeted therapy

Programmed death ligand 1 (PD-L1) is an important immunosuppressive molecule, which can down-regulate the immune system’s response to avoid autoimmune diseases [137, 138] (Fig. 7c). PD-L1 is expressed in a variety of immune and cancer cells. By interacting with PD-1 on T cells, PD-L1 inhibits the activity and growth of T cells, promotes the exhaustion of T cells, and induces apoptosis of activated T cells to help tumor cells to escape host immunity [139, 140]. Therefore, inhibiting the interaction between PD-1 and PD-L1 through inhibitors can allow T cells to kill and eliminate tumor cells normally, a treatment strategy that is effective for anti-tumor immunity. Cyranoski et al. conduct the first CRISPR human trial to treat patients with metastatic non-small cell lung cancer who had failed to respond to chemotherapy, radiation, and other therapies with CRISPR-edited T cells (knockout PD-1 gene) [141, 142]. They recently published the results of the latest trial demonstrating the safety and feasibility of CRISPR gene-edited T cells targeting PD-1 in a cohort of patients with advanced lung cancer(NCT02793856) [143]. Besides, there are clinical trials (NCT02867345, NCT02863913, NCT04417764, NCT03081715, NCT02867332) of CRISPR-mediated PD-1 gene knockout in patients with prostate cancer, bladder cancer, hepatocellular Carcinoma, advanced esophageal cancer and metastatic renal cell carcinoma associated with treatment [144]. Additionally, it is reported that destroying PD-1 enhances the anti-tumor activity of CAR-T cells against hepatocellular carcinoma in vivo, and improves the persistence and infiltration of CAR T cells in tumors [145]. He et al. delivered CRISPR/Cas9 plasmids to the tumor nucleus through the use of an Aptamer/peptide-functionalized vector to knockout the β-catenin, thus downregulating the expression of PD-L1 on tumor cells. They found that the PD-L1 mediated immune escape and immunosuppression of cancer was reversed [146]. These studies provided strategies to reverse tumor immune escape and immunosuppression. The developed genome-editing delivery system has broad research and application prospects for cancer treatment.

### Combating carcinogenic virus infection

The CRISPR/Cas9 system has an antiviral role in bacterial adaptive immunity and thus great potential for the defense and clearance of infected viruses [147]. Carcinogenic viral infections are one of the causes of cancer and commonly include hepatitis B virus (HBV) and hepatitis C virus (HCV) in liver cancer, human papillomavirus (HPV) in laryngeal cancer [126], and Epstein-Barr virus (EBV) in nasopharyngeal carcinoma, Hodgkin’s lymphoma, and Burkitt’s lymphoma [148]. The use of Cas9-sgRNA, which can specifically recognize the viral genome, directly targets oncogenic viral genes or genes required to maintain viral replication. This results in mutations in the viral genome and suppression of oncogenic viral gene expression thereby inducing cancer cell death. Consequently, fighting viral infection and eliminating cancer through CRISPR/Cas technology provides new ideas for the treatment of cancers associated with viral infection (Fig. 7d).

The occurrence of cervical cancer is mainly caused by HPV. HPV-related tumors are attributed to HPV E6 and E7 proteins, which are involved in the malignant transformation of cervical cancer and the maintenance of the malignant phenotype [149]. In HPV16 and HPV-18 positive cervical cancer cells, knockdown of the E6 and E7 genes with CRISPR/Cas9 restored cellular tumor suppressor p53 and retinoblastoma (Rb) protein levels, resulting in cell death and apoptosis [150]. Similar results were also observed when the HPV-16 E6 and E7 genes were knockdown in a nude mouse model of cervical cancer cells, and tumor growth was inhibited [151].

The development of hepatocellular carcinoma (HCC) is closely related to HBV infection of hepatocytes, in which HBV covalent closed-loop DNA (cccDNA) plays a crucial role [152]. Therefore, the removal of cccDNA from hepatocytes is necessary to cure HBV infection. A number of studies have demonstrated that CRISPR/Cas9-mediated HBV DNA editing can effectively reduce cccDNA in cells and mouse models and inhibit viral production [153–156]. Recently, using CRISPR/Cas9 technology, researchers found that the lncRNA PCNAP1 enhances HBV replication by regulating miR-154/PCNA/HBV cccDNA signaling, explaining the mechanism of the effect of lncRNAs on HBV replication and HCC [157]. Besides, the targeted cut mouse p53 and Pten genes were delivered to the liver tissue of adult HBV transgenic mice through the CRISPR/Cas9 system, and tumors were found in the liver of the mice. This observation proved that CRISPR/Cas9-mediated somatic p53 and Pten mutations can accelerate the occurrence of primary hepatocellular carcinoma in adult HBV transgenic mice [158]. HCV can also promote the development of HCC. Price et al. designed *Francisella novicida* Cas9 (FnCas9) to precisely target the viral RNA genome of HCV through RNA-guided RNA recognition, to reduce the production of viral proteins and inhibit HCV infection [159]. Since FnCas9 can target both RNA and DNA, this multifunctional endonuclease may be able to fight multiple types of viruses at the same time, such as HBV and HCV.

EBV is related to a variety of human malignancies, and studies have used the CRISPR/Cas9 system to target EBV. When using Cas9/gRNA transfected Burkitt lymphoma human cells to target EBNA-1, LMP-1, or EBNA-3C genes, the cell proliferation and the viral load are significantly reduced [160] and almost a complete clearance of the latent EBV infection occurs [161]. Moreover, the researchers conducted a human genome-wide CRISPR/Cas9 screen in Burkitt’s lymphoma B cells to systematically analyze the host factors regulating the EBV proliferative infection cycle for the first time and deeply characterized the molecular mechanism of EBV switching from latent infection to viral proliferative infection cycle. Multiple drug therapeutic targets were identified, which laid the foundation for tumor cell-transient immunotherapy targeting EBV infection [162]. These studies proved the potential of CRISPR/Cas9 in preventing and treating cancer virus infections.

## Application of CRISPR/Cas9 in cancer modeling

CRISPR/Cas9 enables in vivo construction of tumor models with multiple genetic mutations to better model complex human diseases (Fig. 7e). Since the emergence of homologous recombination or random transgenic integration, transgenic mice have been the gold standard for cancer modeling studies [163]. The use of laboratory mice to mimic human cancers through remodeling (xenografting) can be used for both basic oncology research to functionally infer cancer genes and for anti-cancer drug screening [164]. The fast and precise CRISPR/Cas9 technology enables researchers to create mouse models of cancer with specific genetic modifications, allowing a more objective study of multistep carcinogenesis. In 2017, Huang et al. created a mouse model of sarcoma using CRISPR/Cas9 technology successfully [165]. Blasco et al. created a mouse model that mimics non-small-cell lung cancers (NSCLC). A specific chromosomal translocation involving the genes Eml4-Alk, which is present in approximately 5–7% of these tumors, was achieved, using a lentiviral CRISPR/Cas9 vector. Employing this system, almost all mice developed lung cancer 8 weeks after the procedure [166]. Disruption of the tumor suppressor genes Pten and P53 in mouse liver by CRISPR/Cas9 has created a mouse model that generates liver tumors with a cancer phenotype similar to that of mice made with conventional Cre-loxP technology [167]. These models allow for the study of potential mechanisms of tumorigenesis and progression while exploring various therapeutic approaches.

Patient-derived xenograft (PDX) animal models can enable exogenous growing of human tumors and provide an indispensable preclinical tool for oncology research [168]. Mice are the most widely used host for human PDX models; nevertheless, the small size of mice limits the growth of xenografts, which in turn affects sample collection and drug evaluation. Thus, the researchers used the CRISPR/Cas9 technique to knockout Rag1, Rag2, and n Il2 in Sprague Dawley (SD) rats to develop a new rat model with significantly impaired lymphoid organ development. The SD-RG rats with severe immunodeficiency overcame the above shortcomings and have successfully been developed into a PDX model of lung squamous cell carcinoma in which the grafts reproduced the histopathological features of the primary tumor in multiple passages. This has great potential to be used as a new model for cancer research [169].

Zebrafish have been used for the study of different types of cancers, such as skin cancer [170, 171] pancreatic cancer [172], breast cancer [173], leukemia [174], glioma [175], and lung cancer [176]. With the help of the CRISPR/Cas9 system, zebrafish has obtained a flexible, cheap, and easy tool in research that can generate mosaic knockout or lines on demand. This will improve the analysis of tumor suppressor genes that are difficult to study and make the development of a more complicated Zebrafish cancer model possible [177]. Ablain et al. used CRISPR technology in their study to create a zebrafish model of genetic SPRED1-deficient mucosal melanoma and found that SPRED1 plays a tumor suppressor role. These findings provide a rationale for MAPK (mitogen-activated protein kinase) inhibition in SPRED1-deficient melanoma and suggest a new zebrafish modeling approach that can be used to rapidly study genetic mutations occurring in human cancers [178]. These emerging modeling approaches could allow for a more efficient investigation of cancer genes in vitro and in vivo.

## Application of CRISPR/Cas system in a new emerging hotspot of oncology

RNA modification mediated by N6-methyladenosine (m6A) influences practically the whole post-transcriptional processes. Emerging evidence reveals that m6A modification is correlated with tumor initiation, proliferation, differentiation, invasion, metastasis, and survival rate [179]. Furthermore, the regulators of m6A modification function as oncogenes or tumor suppressors in various cancers [180, 181]. However, previous RNA biology methods cannot distinguish the effect of individual m6A modifications. Recently, based on the CRISPR/Cas9 technology, Liu et al. developed a robust approach that enables m6A accession and erasure at the specific site via recognition of homologous sequence from cellular RNAs. They engineered fusions of dCas9 with m6A methyltransferases METTL3 and METTL14 and found that the resultant m6A ‘writers’ enable comparison of the action of single-site methylation by distinct mRNA regions. In a further study, they used m6A ‘erasers’ via fusing CRISPR/Cas9 with ALKBH5 or FTO to achieve RNAs demethylation in a specific site [182]. Latest, Wilson et al. developed a targeted RNA methylation (TRM) system based on the RNA-targeting ability of CRISPR/Cas13 in combination with RNA methyltransferases generated m6A. They indicated that site-specific incorporation of m6A into different cellular compartments is guided by fusion of dCas13 with the METTL3 methyltransferase domain. They also established that the cytoplasmic localization of the METTL3:METTL14 methyltransferase complexes domain and cytoplasm-localized fusions with a modified METTL3:METTL14 methyltransferase complex can direct site-specific m6A incorporation in distinct cellular compartments [183]. The TRM is a targeted epigenome engineering tool used to reveal and analyze the functions of individual m6A modifications. Utilization of these tools will facilitate mechanistic understanding and clinical trials of malignant tumors in a way of epitranscriptome.

Long non-coding RNAs (lncRNA) are considered to be the main “dark matter” in the genome. Numerous studies have shown that these RNAs may participate in a series of important physiological activities in cells and are also closely related to the initiation and progression of cancers [184]. The interaction between RNA binding proteins (RBP) and lncRNAs determines the function and fate of RNA molecules. Therefore, accurate identification of lncRNA interacting proteins in living cells can help us in revealing the molecular mechanism of some complex human diseases better [185]. Recently, using CRISPR/CasRx-based RNA targeting and proximity labeling to characterize specific long non-coding RNAs (lncRNAs) binding proteins in primary cells, Yi et al. established the CRISPR-assisted RNA-protein interaction detection method (CARPID). The detection method was applied in nuclear lncRNA XIST and captured a range of known interacting proteins and plentiful unidentified binding proteins [186]. This tool can be used to detect the RNA-binding proteins that are significantly involved in tumorigenesis, and these protein molecules may serve as drug targets for treating tumor-related diseases.

The CRISPR/Cas adaptive immune defense systems in some cases can protect against specific sequences of foreign DNA or RNA. However, phages have not been eliminated by the evolution of defense systems in the bacterial host, suggesting that there are genes in some phages that encode antagonistic bacterial CRISPR/Cas immune system products. Expectedly, recent studies have discovered a group of bacteriophage proteins, anti-CRISPRs (Acrs), that can inactivate certain CRISPR systems [187, 188]. Nakamura et al. evaluated the activity of a panel of Acrs in mammalian cells using both CRISPRi and CRISPRa. Their results clearly suggested that AcrIIA4 is a potent regulator of (d) Cas9 activity in various cell types. Moreover, AcrIIA4 benefits from its small size which enables easy incorporation into a range of environments and it can efficiently inhibit CRISPR activity when it is fused to other gene products via abounding linkers [189]. These studies indicated that anti-CRISPR proteins could act as preferable tools for the generation of more advanced dynamic control over gene regulation by regulating CRISPR activity in cancer research, thus providing more approaches for overcoming the complex features of cancer.

## Conclusions and perspectives

In just a few years, the CRISPR/Cas9 has emerged and advanced rapidly as a stable, efficient, simple, and extensively used gene editing technology. Actually, the CRISPR/Cas9 has fundamentally impacted many fields, such as agriculture, biotech, and biomedicine, but no field has felt a more profound impact than cancer research as evidenced by the accumulating data in the fast-growing publications. The capacity to implement the genome rapidly and accurately opened the windows for a new and more elaborate outlook on the mechanisms of tumorigenesis and progression. More importantly, CRISPR/cas9 gene editing technology hold big promise for cancer therapy. However, several challenges remain before this technology can be used in clinical treatment of cancer safely and efficiently.

Firstly, the off-target effect of CRISPR/Cas9 gene editing technology has always been a major concern. Therefore, improvement of the specificity and the tools for off-target detection is required for safe therapeutic uses of CRISPR/Cas9. Many efforts have been made for increasing specificity, including development of more prioritizing sgRNA designer by integrating of multiple factors [190], discovery and use of more specific Cas9 variants, limiting the time of CRISPR/Cas9 activity, use of inducible Cas9 variants, and use of anti-CRISPR proteins [191]. Furthermore, techniques such as GUIDE-Seq [192], CIRCLE-Seq [193], and CHANGE-seq [194] that can detect low-frequency mutations have been developed. Further studies are needed to fully understand the principles that govern CRISPR/Cas9 specificity, and to improve off-target detection sensitivity.

Secondly, on-target mutagenesis was occurred frequently in double-strand breaks induced by single-guided RNA/Cas9, such as large deletions (over many kilobases) and complex genomic rearrangements at the targeted sites, thus will elicit long-range transcriptional consequences and may have pathogenic consequences [195] . Therefore, the technology of precise spatiotemporal control of CRISPR/Cas9 activity in cells and complex conditions will be beneficial, such as cell-specific promoters, small molecule activation/inhibition, bioresponsive delivery carriers and optical/ultrasonic/thermal/magnetic activation of the CRISPR/Cas9 system [196]. In addition, it is necessary to perform comprehensive genomic analysis to identify cells with normal genomes before clinical applications.

Thirdly, efficient, safe and targetable delivery of the CRISPR/Cas9 system in vivo is also a huge challenge for clinical application. To overcome this problem, novel delivery strategies and control mechanisms is required. Fortunately, a series of viral and nonviral delivery systems were developed for gene editing in diverse tissues, and these methods all show certain advantages and disadvantages [197–199]. Recently, multifunctional nanoparticles with tumor pH response, active EGFR targeting, and nuclear localization provided new idea for overcoming the delivery problem of the CRISPR/Cas system [200]. Looking ahead, a delivery system that enable to deliver the CRISPR/Cas9 components for tissue- and cell-type-specific gene editing with safety and efficiency is ideal for clinical translation.

Fourthly, another challenge for CRISPR/Cas9 application is the human body’s immune response to the bacteria-derived Cas9 protein. Charlesworth et al. detected antibodies against Cas9 in human serum by ELISA (enzyme-linked immunosorbent assay). Results showed that both SaCas9 and SpCas9 antibodies were existed in 78 and 58% of subjects, respectively. Furthermore, they also found anti-SaCas9 and anti-SpCas9 T cells in 78 and 67% of subjects, respectively [201]. These data indicated that there are preexisting humoral and cell-mediated adaptive immune responses to Cas9 in humans that may compromise efficiency of gene editing. Therefore, optimizing vector, dose, administration route, and immune suppression are potential approaches to perfect the CRISPR/Cas9 gene editing in vivo.

Finally, the DNA double-strand break caused by CRISPR/Cas9 can activate the p53 pathway, induce a p53-mediated DNA damage response and cell cycle arrest, thus leading to failure of gene editing [202, 203] . Nevertheless, recent studies revealed that although Cas9-induced p53 pathway activation alters cellular sensitivity to both genetic and chemical perturbations [204], careful experimental design and thorough data analysis made it is possible to get useful results even in cells with functional p53 protein [205]. In addition, it has been demonstrated that the single Cas9 nickase approach, which does not rely on double-stranded DNA breaks, is expected to circumvent this risk [206]. To usher a golden age of the CRISPR/Cas technology in cancer research, diagnosis and treatment, continuous efforts are needed to overcome the above challenges in future.

## Data Availability

Not applicable.

## Abbreviations

CRISPR/Cas9: Clustered regularly interspaced short palindromic repeats/CRISPR-associated nuclease9
ZFN: Zinc finger endonuclease
TALEN: Transcription activator-like effector nuclease
sgRNA: Single-stranded guide RNA
PAM: Protospacer adjacent motif
DSB: Double-strand break
NHEJ: Non-homologous end-joining
HDR: Homology-directed repair
dCas9: Endonuclease-deficient Cas9
CRISPRa: CRISPR activation
CRISPRi: CRISPR inhibition
ABE: Adenine bases Editor
nCATS: Nanopore Cas9-targeted sequence
vfCRISPR: Very fast CRISPR
SpCas9: *Streptococcus pyogenes* Cas9
DETECTR: DNA Endonuclease Targeted CRISPR Trans Reporter
REPAIR: RNA Editing for Programmable Adenosine to Inosine Replacement
ssRNA: Single-stranded RNA
SHERLOCK: Specific High Sensitivity Enzymatic Reporter Unlocking
CARVER: Cas13-assisted restriction of virus expression and readout
PC: Pancreatic cancer
TNBC: Triple-negative breast cancer
TKI: Tyrosine kinase inhibitor
AML: Acute myeloid leukemia
OCSC: Ovarian cancer stem cell
CSC: Cancer stem cell
GecKO: Genome-wide CRISPR/Cas9 knockout
HCC: Hepatocellular carcinoma
ACT: Adoptive cell therapy
CAPTURE: CRISPR affinity purification in situ of regulatory element
FISH: Fluorescence in situ hybridization
CART: Chimeric antigen receptor T cell
ALL: Acute lymphoblastic leukemia
CRS: Cytokine release syndrome
HBV: Hepatitis B virus
HCV: Hepatitis C virus
HPV: Human papillomavirus
EBV: Epstein-Barr virus
FnCas9: *Francisella novicida* Cas9
NSCLC: Non-small-cell lung cancer
PDX: Patient-derived xenograft
TRM: Targeted RNA methylation
lncRNA: Long non-coding RNA
CARPID: CRISPR-assisted RNA-protein interaction detection
GUIDE-Seq: Genome-wide, unbiased identification of DSBs enabled by sequencing
CIRCLE-Seq: Circularization for in vitro reporting of cleavage effects by sequencing
CHANGE-seq: Circularization for high-throughput analysis of nuclease genome-wide effects by sequencing

## References

[1] Sung H, Ferlay J, Siegel RL, Laversanne M, Soerjomataram I, Jemal A, et al. Global cancer statistics 2020: GLOBOCAN estimates of incidence and mortality worldwide for 36 cancers in 185 countries. CA Cancer J Clin. 2021;71:209–49. 10.3322/caac.21660 33538338

[2] Stupp R, Hegi ME, Mason WP, van den Bent MJ, Taphoorn MJ, Janzer RC (2009). Effects of radiotherapy with concomitant and adjuvant temozolomide versus radiotherapy alone on survival in glioblastoma in a randomised phase III study: 5-year analysis of the EORTC-NCIC trial. Lancet Oncol.

[3] Garraway LA, Lander ES (2013). Lessons from the cancer genome. Cell..

[4] Sánchez-Rivera FJ, Jacks T (2015). Applications of the CRISPR-Cas9 system in cancer biology. Nat Rev Cancer.

[5] Pon JR, Marra MA (2015). Driver and passenger mutations in cancer. Annu Rev Pathol.

[6] Shen P, Jing Y, Zhang R, Cai MC, Ma P, Chen H (2018). Comprehensive genomic profiling of neuroendocrine bladder cancer pinpoints molecular origin and potential therapeutics. Oncogene..

[7] Carroll D (2011). Genome engineering with zinc-finger nucleases. Genetics..

[8] Joung JK, Sander JD (2013). TALENs: a widely applicable technology for targeted genome editing. Nat Rev Mol Cell Biol.

[9] Zhang F, Wen Y, Guo X (2014). CRISPR/Cas9 for genome editing: progress, implications and challenges. Hum Mol Genet.

[10] Rupp LJ, Schumann K, Roybal KT, Gate RE, Ye CJ, Lim WA (2017). CRISPR/Cas9-mediated PD-1 disruption enhances anti-tumor efficacy of human chimeric antigen receptor T cells. Sci Rep.

[11] Zuckermann M, Hovestadt V, Knobbe-Thomsen CB, Zapatka M, Northcott PA, Schramm K (2015). Somatic CRISPR/Cas9-mediated tumour suppressor disruption enables versatile brain tumour modelling. Nat Commun.

[12] Horvath P, Barrangou R (2010). CRISPR/Cas, the immune system of bacteria and archaea. Science..

[13] Garneau JE, Dupuis MÈ, Villion M, Romero DA, Barrangou R, Boyaval P (2010). The CRISPR/Cas bacterial immune system cleaves bacteriophage and plasmid DNA. Nature..

[14] Cong L, Ran FA, Cox D, Lin S, Barretto R, Habib N (2013). Multiplex genome engineering using CRISPR/Cas systems. Science..

[15] Jinek M, Chylinski K, Fonfara I, Hauer M, Doudna JA, Charpentier E (2012). A programmable dual-RNA-guided DNA endonuclease in adaptive bacterial immunity. Science..

[16] Pawelczak KS, Gavande NS, VanderVere-Carozza PS, Turchi JJ (2018). Modulating DNA repair pathways to improve precision genome engineering. ACS Chem Biol.

[17] Ishino Y, Shinagawa H, Makino K, Amemura M, Nakata A (1987). Nucleotide sequence of the iap gene, responsible for alkaline phosphatase isozyme conversion in Escherichia coli, and identification of the gene product. J Bacteriol.

[18] Jansen R, Embden JD, Gaastra W, Schouls LM (2002). Identification of genes that are associated with DNA repeats in prokaryotes. Mol Microbiol.

[19] Mojica FJ, Díez-Villaseñor C, García-Martínez J, Soria E (2005). Intervening sequences of regularly spaced prokaryotic repeats derive from foreign genetic elements. J Mol Evol.

[20] Pourcel C, Salvignol G, Vergnaud G (2005). CRISPR elements in Yersinia pestis acquire new repeats by preferential uptake of bacteriophage DNA, and provide additional tools for evolutionary studies. Microbiology (Reading).

[21] Bolotin A, Quinquis B, Sorokin A, Ehrlich SD (2005). Clustered regularly interspaced short palindrome repeats (CRISPRs) have spacers of extrachromosomal origin. Microbiology (Reading).

[22] Barrangou R, Fremaux C, Deveau H, Richards M, Boyaval P, Moineau S (2007). CRISPR provides acquired resistance against viruses in prokaryotes. Science..

[23] Brouns SJ, Jore MM, Lundgren M, Westra ER, Slijkhuis RJ, Snijders AP (2008). Small CRISPR RNAs guide antiviral defense in prokaryotes. Science..

[24] Deltcheva E, Chylinski K, Sharma CM, Gonzales K, Chao Y, Pirzada ZA (2011). CRISPR RNA maturation by trans-encoded small RNA and host factor RNase III. Nature..

[25] Mali P, Yang L, Esvelt KM, Aach J, Guell M, DiCarlo JE (2013). RNA-guided human genome engineering via Cas9. Science..

[26] Qi LS, Larson MH, Gilbert LA, Doudna JA, Weissman JS, Arkin AP (2013). Repurposing CRISPR as an RNA-guided platform for sequence-specific control of gene expression. Cell..

[27] Maeder ML, Linder SJ, Cascio VM, Fu Y, Ho QH, Joung JK (2013). CRISPR RNA-guided activation of endogenous human genes. Nat Methods.

[28] Gilbert LA, Larson MH, Morsut L, Liu Z, Brar GA, Torres SE (2013). CRISPR-mediated modular RNA-guided regulation of transcription in eukaryotes. Cell..

[29] Komor AC, Kim YB, Packer MS, Zuris JA, Liu DR (2016). Programmable editing of a target base in genomic DNA without double-stranded DNA cleavage. Nature..

[30] Gaudelli NM, Komor AC, Rees HA, Packer MS, Badran AH, Bryson DI (2017). Programmable base editing of a•T to G•C in genomic DNA without DNA cleavage. Nature..

[31] Doman JL, Raguram A, Newby GA, Liu DR (2020). Evaluation and minimization of Cas9-independent off-target DNA editing by cytosine base editors. Nat Biotechnol.

[32] Miller SM, Wang T, Randolph PB, Arbab M, Shen MW, Huang TP (2020). Continuous evolution of SpCas9 variants compatible with non-G PAMs. Nat Biotechnol.

[33] Gilpatrick T, Lee I, Graham JE, Raimondeau E, Bowen R, Heron A (2020). Targeted nanopore sequencing with Cas9-guided adapter ligation. Nat Biotechnol.

[34] Liu Y, Zou RS, He S, Nihongaki Y, Li X, Razavi S (2020). Very fast CRISPR on demand. Science..

[35] Mojica F, Díez-Villaseñor C, García-Martínez J, Almendros C (2009). Short motif sequences determine the targets of the prokaryotic CRISPR defence system. Microbiology (Reading)..

[36] Kleinstiver BP, Prew MS, Tsai SQ, Topkar VV, Nguyen NT, Zheng Z (2015). Engineered CRISPR-Cas9 nucleases with altered PAM specificities. Nature..

[37] Nishimasu H, Cong L, Yan WX, Ran FA, Zetsche B, Li Y (2015). Crystal structure of Staphylococcus aureus Cas9. Cell..

[38] Ran FA, Cong L, Yan WX, Scott DA, Gootenberg JS, Kriz AJ (2015). In vivo genome editing using Staphylococcus aureus Cas9. Nature..

[39] Hu JH, Miller SM, Geurts MH, Tang W, Chen L, Sun N (2018). Evolved Cas9 variants with broad PAM compatibility and high DNA specificity. Nature..

[40] Nishimasu H, Shi X, Ishiguro S, Gao L, Hirano S, Okazaki S (2018). Engineered CRISPR-Cas9 nuclease with expanded targeting space. Science..

[41] Walton RT, Christie KA, Whittaker MN, Kleinstiver BP (2020). Unconstrained genome targeting with near-PAMless engineered CRISPR-Cas9 variants. Science..

[42] Morsy SG, Tonne JM, Zhu Y, Lu B, Budzik K, Krempski JW (2017). Divergent susceptibilities to AAV-SaCas9-gRNA vector-mediated genome-editing in a single-cell-derived cell population. BMC Res Notes.

[43] Koo T, Lu-Nguyen NB, Malerba A, Kim E, Kim D, Cappellari O (2018). Functional Rescue of Dystrophin Deficiency in mice caused by Frameshift mutations using campylobacter jejuni Cas9. Mol Ther.

[44] Fujii W, Ito H, Kanke T, Ikeda A, Sugiura K, Naito K (2019). Generation of genetically modified mice using SpCas9-NG engineered nuclease. Sci Rep.

[45] Casini A, Olivieri M, Petris G, Montagna C, Reginato G, Maule G (2018). A highly specific SpCas9 variant is identified by in vivo screening in yeast. Nat Biotechnol.

[46] Chen JS, Ma E, Harrington LB, Da Costa M, Tian X, Palefsky JM (2018). CRISPR-Cas12a target binding unleashes indiscriminate single-stranded DNase activity. Science..

[47] Zetsche B, Gootenberg JS, Abudayyeh OO, Slaymaker IM, Makarova KS, Essletzbichler P (2015). Cpf1 is a single RNA-guided endonuclease of a class 2 CRISPR-Cas system. Cell..

[48] Shmakov S, Abudayyeh OO, Makarova KS, Wolf YI, Gootenberg JS, Semenova E (2015). Discovery and functional characterization of diverse class 2 CRISPR-Cas systems. Mol Cell.

[49] Yang H, Gao P, Rajashankar KR, Patel DJ (2016). PAM-Dependent Target DNA Recognition and Cleavage by C2c1 CRISPR-Cas Endonuclease. Cell.

[50] Koonin EV, Makarova KS, Zhang F (2017). Diversity, classification and evolution of CRISPR-Cas systems. Curr Opin Microbiol.

[51] Teng F, Cui T, Feng G, Guo L, Xu K, Gao Q (2018). Repurposing CRISPR-Cas12b for mammalian genome engineering. Cell Discov.

[52] Broughton JP, Deng X, Yu G, Fasching CL, Servellita V, Singh J (2020). CRISPR-Cas12-based detection of SARS-CoV-2. Nat Biotechnol.

[53] Mahas A, Neal Stewart C, Mahfouz MM (2018). Harnessing CRISPR/Cas systems for programmable transcriptional and post-transcriptional regulation. Biotechnol Adv.

[54] Abudayyeh OO, Gootenberg JS, Konermann S, Joung J, Slaymaker IM, Cox DB (2016). C2c2 is a single-component programmable RNA-guided RNA-targeting CRISPR effector. Science.

[55] East-Seletsky A, O'Connell MR, Knight SC, Burstein D, Cate JH, Tjian R (2016). Two distinct RNase activities of CRISPR-C2c2 enable guide-RNA processing and RNA detection. Nature..

[56] Granados-Riveron JT, Aquino-Jarquin G (2018). CRISPR-Cas13 precision Transcriptome engineering in Cancer. Cancer Res.

[57] Freije CA, Myhrvold C, Boehm CK, Lin AE, Welch NL, Carter A (2019). Programmable Inhibition and Detection of RNA Viruses Using Cas13. Mol Cell.

[58] Abudayyeh OO, Gootenberg JS, Essletzbichler P, Han S, Joung J, Belanto JJ (2017). RNA targeting with CRISPR-Cas13. Nature..

[59] Gootenberg JS, Abudayyeh OO, Lee JW, Essletzbichler P, Dy AJ, Joung J (2017). Nucleic acid detection with CRISPR-Cas13a/C2c2. Science..

[60] Nguyen TM, Zhang Y, Pandolfi PP (2020). Virus against virus: a potential treatment for 2019-nCov (SARS-CoV-2) and other RNA viruses. Cell Res.

[61] Vogelstein B, Kinzler KW (1993). The multistep nature of cancer. Trends Genet.

[62] Liu Y, Hu X, Han C, Wang L, Zhang X, He X (2015). Targeting tumor suppressor genes for cancer therapy. Bioessays..

[63] Hanahan D, Weinberg RA (2011). Hallmarks of cancer: the next generation. Cell..

[64] Jiang C, Meng L, Yang B, Luo X (2020). Application of CRISPR/Cas9 gene editing technique in the study of cancer treatment. Clin Genet.

[65] Li W, Cho MY, Lee S, Jang M, Park J, Park R (2019). CRISPR-Cas9 mediated CD133 knockout inhibits colon cancer invasion through reduced epithelial-mesenchymal transition. PLoS One.

[66] Yan J, Jia Y, Chen H, Chen W, Zhou X (2019). Long non-coding RNA PXN-AS1 suppresses pancreatic cancer progression by acting as a competing endogenous RNA of miR-3064 to upregulate PIP4K2B expression. J Exp Clin Cancer Res.

[67] Koo T, Yoon AR, Cho HY, Bae S, Yun CO, Kim JS (2017). Selective disruption of an oncogenic mutant allele by CRISPR/Cas9 induces efficient tumor regression. Nucleic Acids Res.

[68] Tang KJ, Constanzo JD, Venkateswaran N, Melegari M, Ilcheva M, Morales JC (2016). Focal adhesion kinase regulates the DNA damage response and its inhibition Radiosensitizes mutant KRAS lung Cancer. Clin Cancer Res.

[69] Liao L, Song M, Li X, Tang L, Zhang T, Zhang L (2017). E3 ubiquitin ligase UBR5 drives the growth and metastasis of triple-negative breast Cancer. Cancer Res.

[70] Chen ML, Chang JH, Yeh KT, Chang YS, Chang JG (2007). Epigenetic changes in tumor suppressor genes, P15, P16, APC-3 and E-cadherin in body fluid. Kaohsiung J Med Sci.

[71] Morris LG, Chan TA (2015). Therapeutic targeting of tumor suppressor genes. Cancer..

[72] Wahiduzzaman M, Karnan S, Ota A, Hanamura I, Murakami H, Inoko A (2019). Establishment and characterization of CRISPR/Cas9-mediated NF2−/− human mesothelial cell line: molecular insight into fibroblast growth factor receptor 2 in malignant pleural mesothelioma. Cancer Sci.

[73] Xu K, Chen G, Li X, Wu X, Chang Z, Xu J (2017). MFN2 suppresses cancer progression through inhibition of mTORC2/Akt signaling. Sci Rep.

[74] Pan WW, Moroishi T, Koo JH, Guan KL (2019). Cell type-dependent function of LATS1/2 in cancer cell growth. Oncogene..

[75] Moses C, Nugent F, Waryah CB, Garcia-Bloj B, Harvey AR, Blancafort P (2019). Activating PTEN tumor suppressor expression with the CRISPR/dCas9 system. Mol Ther Nucleic Acids..

[76] Artegiani B, van Voorthuijsen L, Lindeboom R, Seinstra D, Heo I, Tapia P (2019). Probing the Tumor Suppressor Function of BAP1 in CRISPR-Engineered Human Liver Organoids. Cell Stem Cell.

[77] Maji S, Panda S, Samal SK, Shriwas O, Rath R, Pellecchia M (2018). Bcl-2 Antiapoptotic family proteins and Chemoresistance in Cancer. Adv Cancer Res.

[78] Bialk P, Wang Y, Banas K, Kmiec EB (2018). Functional gene knockout of NRF2 increases Chemosensitivity of human lung Cancer A549 cells in vitro and in a Xenograft mouse model. Mol Ther Oncolytics.

[79] Gao W, Zhang Y, Luo H, Niu M, Zheng X, Hu W (2020). Targeting SKA3 suppresses the proliferation and chemoresistance of laryngeal squamous cell carcinoma via impairing PLK1-AKT axis-mediated glycolysis. Cell Death Dis.

[80] Chen X, Sun X, Guan J, Gai J, Xing J, Fu L (2017). Rsf-1 influences the sensitivity of non-small cell lung Cancer to paclitaxel by regulating NF-κB pathway and its downstream proteins. Cell Physiol Biochem.

[81] Heyza JR, Lei W, Watza D, Zhang H, Chen W, Back JB (2019). Identification and characterization of synthetic viability with ERCC1 deficiency in response to Interstrand crosslinks in lung Cancer. Clin Cancer Res.

[82] Yu J, Zhou J, Xu F, Bai W, Zhang W (2018). High expression of Aurora-B is correlated with poor prognosis and drug resistance in non-small cell lung cancer. Int J Biol Markers.

[83] Pavlova NN, Thompson CB (2016). The emerging hallmarks of Cancer metabolism. Cell Metab.

[84] Li Z, Zhang H (2016). Reprogramming of glucose, fatty acid and amino acid metabolism for cancer progression. Cell Mol Life Sci.

[85] Yasuda M, Miyazawa M, Fujita M, Kajiwara H, Iida T, Hirasawa T (2008). Expression of hypoxia inducible factor-1alpha (HIF-1alpha) and glucose transporter-1 (GLUT-1) in ovarian adenocarcinomas: difference in hypoxic status depending on histological character. Oncol Rep.

[86] Pez F, Dayan F, Durivault J, Kaniewski B, Aimond G, Le Provost GS (2011). The HIF-1-inducible lysyl oxidase activates HIF-1 via the Akt pathway in a positive regulation loop and synergizes with HIF-1 in promoting tumor cell growth. Cancer Res.

[87] Wu XH, Chen SP, Mao JY, Ji XX, Yao HT, Zhou SH (2013). Expression and significance of hypoxia-inducible factor-1α and glucose transporter-1 in laryngeal carcinoma. Oncol Lett.

[88] Lu ZJ, Yu Q, Zhou SH, Fan J, Shen LF, Bao YY (2019). Construction of a GLUT-1 and HIF-1α gene knockout cell model in HEp-2 cells using the CRISPR/Cas9 technique. Cancer Manag Res.

[89] Gallipoli P, Giotopoulos G, Tzelepis K, Costa A, Vohra S, Medina-Perez P (2018). Glutaminolysis is a metabolic dependency in FLT3ITD acute myeloid leukemia unmasked by FLT3 tyrosine kinase inhibition. Blood..

[90] Kanarek N, Keys HR, Cantor JR, Lewis CA, Chan SH, Kunchok T (2018). Histidine catabolism is a major determinant of methotrexate sensitivity. Nature..

[91] Papaccio F, Paino F, Regad T, Papaccio G, Desiderio V, Tirino V (2017). Concise review: Cancer cells, Cancer stem cells, and Mesenchymal stem cells: influence in Cancer development. Stem Cells Transl Med.

[92] Zomer A, Ellenbroek SI, Ritsma L, Beerling E, Vrisekoop N, Van Rheenen J (2013). Intravital imaging of cancer stem cell plasticity in mammary tumors. Stem Cells.

[93] Noh KH, Kim BW, Song KH, Cho H, Lee YH, Kim JH (2012). Nanog signaling in cancer promotes stem-like phenotype and immune evasion. J Clin Invest.

[94] Chang C, Lee SO, Yeh S, Chang TM (2014). Androgen receptor (AR) differential roles in hormone-related tumors including prostate, bladder, kidney, lung, breast and liver. Oncogene..

[95] Ling K, Jiang L, Liang S, Kwong J, Yang L, Li Y (2018). Nanog interaction with the androgen receptor signaling axis induce ovarian cancer stem cell regulation: studies based on the CRISPR/Cas9 system. J Ovarian Res.

[96] Yang F, Cui P, Lu Y, Zhang X (2019). Requirement of the transcription factor YB-1 for maintaining the stemness of cancer stem cells and reverting differentiated cancer cells into cancer stem cells. Stem Cell Res Ther.

[97] Krausova M, Korinek V (2014). Wnt signaling in adult intestinal stem cells and cancer. Cell Signal.

[98] Zhan T, Ambrosi G, Wandmacher AM, Rauscher B, Betge J, Rindtorff N (2019). MEK inhibitors activate Wnt signalling and induce stem cell plasticity in colorectal cancer. Nat Commun.

[99] Hwang JH, Yoon J, Cho YH, Cha PH, Park JC, Choi KY (2020). A mutant KRAS-induced factor REG4 promotes cancer stem cell properties via Wnt/β-catenin signaling. Int J Cancer.

[100] Bester AC, Lee JD, Chavez A, Lee YR, Nachmani D, Vora S (2018). An Integrated Genome-wide CRISPRa Approach to Functionalize lncRNAs in Drug Resistance. Cell.

[101] Shalem O, Sanjana NE, Hartenian E, Shi X, Scott DA, Mikkelson T (2014). Genome-scale CRISPR-Cas9 knockout screening in human cells. Science..

[102] Chen S, Sanjana NE, Zheng K, Shalem O, Lee K, Shi X (2015). Genome-wide CRISPR screen in a mouse model of tumor growth and metastasis. Cell..

[103] Totoki Y, Tatsuno K, Covington KR, Ueda H, Creighton CJ, Kato M (2014). Trans-ancestry mutational landscape of hepatocellular carcinoma genomes. Nat Genet.

[104] Schwartzentruber J, Korshunov A, Liu XY, Jones DT, Pfaff E, Jacob K (2012). Driver mutations in histone H3.3 and chromatin remodelling genes in paediatric glioblastoma. Nature..

[105] Liang J, Zhao H, Diplas BH, Liu S, Liu J, Wang D (2020). Genome-wide CRISPR-Cas9 screen reveals selective vulnerability of ATRX-mutant cancers to WEE1 inhibition. Cancer Res.

[106] Wei J, Long L, Zheng W, Dhungana Y, Lim SA, Guy C (2019). Targeting REGNASE-1 programs long-lived effector T cells for cancer therapy. Nature..

[107] Zhu S, Li W, Liu J, Chen CH, Liao Q, Xu P (2016). Genome-scale deletion screening of human long non-coding RNAs using a paired-guide RNA CRISPR-Cas9 library. Nat Biotechnol.

[108] Liu Y, Cao Z, Wang Y, Guo Y, Xu P, Yuan P, et al. Genome-wide screening for functional long noncoding RNAs in human cells by Cas9 targeting of splice sites. Nat Biotechnol. 2018;36:1203–10. 10.1038/nbt.4283 30395134

[109] Gilbert LA, Horlbeck MA, Adamson B, Villalta JE, Chen Y, Whitehead EH (2014). Genome-scale CRISPR-mediated control of gene repression and activation. Cell..

[110] Liu SJ, Horlbeck MA, Cho SW, Birk HS, Malatesta M, He D, et al. CRISPRi-based genome-scale identification of functional long noncoding RNA loci in human cells. Science. 2017;355:aah7111. 10.1126/science.aah7111 PMC5394926 27980086

[111] Jost M, Chen Y, Gilbert LA, Horlbeck MA, Krenning L, Menchon G (2017). Combined CRISPRi/a-Based Chemical Genetic Screens Reveal that Rigosertib Is a Microtubule-Destabilizing Agent. Mol Cell.

[112] Raffeiner P, Hart JR, García-Caballero D, Bar-Peled L, Weinberg MS, Vogt PK (2020). An MXD1-derived repressor peptide identifies noncoding mediators of MYC-driven cell proliferation. Proc Natl Acad Sci U S A.

[113] Konermann S, Brigham MD, Trevino AE, Joung J, Abudayyeh OO, Barcena C (2015). Genome-scale transcriptional activation by an engineered CRISPR-Cas9 complex. Nature..

[114] Kurata M, Yamamoto K, Moriarity BS, Kitagawa M, Largaespada DA (2018). CRISPR/Cas9 library screening for drug target discovery. J Hum Genet.

[115] Wang G, Chow RD, Bai Z, Zhu L, Errami Y, Dai X (2019). Multiplexed activation of endogenous genes by CRISPRa elicits potent antitumor immunity. Nat Immunol.

[116] Qi F, Tan B, Ma F, Zhu B, Zhang L, Liu X (2019). A synthetic light-switchable system based on CRISPR Cas13a regulates the expression of LncRNA MALAT1 and affects the malignant phenotype of bladder Cancer cells. Int J Biol Sci.

[117] Wang Q, Liu X, Zhou J, Yang C, Wang G, Tan Y (2019). The CRISPR-Cas13a Gene-Editing System Induces Collateral Cleavage of RNA in Glioma Cells. Adv Sci (Weinh).

[118] Fujita T, Fujii H (2015). Isolation of specific genomic regions and identification of associated molecules by engineered DNA-binding molecule-mediated chromatin immunoprecipitation (enChIP) using CRISPR. Methods Mol Biol.

[119] Fujita T, Fujii H (2014). Identification of proteins associated with an IFNγ-responsive promoter by a retroviral expression system for enChIP using CRISPR. PLoS One.

[120] Liu X, Zhang Y, Chen Y, Li M, Zhou F, Li K (2017). In Situ Capture of Chromatin Interactions by Biotinylated dCas9. Cell.

[121] Zhou Y, Wang P, Tian F, Gao G, Huang L, Wei W (2017). Painting a specific chromosome with CRISPR/Cas9 for live-cell imaging. Cell Res.

[122] Ma H, Naseri A, Reyes-Gutierrez P, Wolfe SA, Zhang S, Pederson T (2015). Multicolor CRISPR labeling of chromosomal loci in human cells. Proc Natl Acad Sci U S A.

[123] Shao S, Zhang W, Hu H, Xue B, Qin J, Sun C (2016). Long-term dual-color tracking of genomic loci by modified sgRNAs of the CRISPR/Cas9 system. Nucleic Acids Res.

[124] Artegiani B, Hendriks D, Beumer J, Kok R, Zheng X, Joore I (2020). Fast and efficient generation of knock-in human organoids using homology-independent CRISPR-Cas9 precision genome editing. Nat Cell Biol.

[125] Rahman N (2014). Mainstreaming genetic testing of cancer predisposition genes. Clin Med (Lond).

[126] Yang D, Shi Y, Tang Y, Yin H, Guo Y, Wen S (2019). Effect of HPV infection on the occurrence and development of laryngeal Cancer: a review. J Cancer.

[127] Qiu XY, Zhu LY, Zhu CS, Ma JX, Hou T, Wu XM (2018). Highly effective and low-cost MicroRNA detection with CRISPR-Cas9. ACS Synth Biol.

[128] Vogelstein B, Papadopoulos N, Velculescu VE, Zhou S, Diaz LA, Kinzler KW (2013). Cancer genome landscapes. Science..

[129] Neelapu SS, Locke FL, Bartlett NL, Lekakis LJ, Miklos DB, Jacobson CA (2017). Axicabtagene Ciloleucel CAR T-cell therapy in refractory large B-cell lymphoma. N Engl J Med.

[130] Fry TJ, Shah NN, Orentas RJ, Stetler-Stevenson M, Yuan CM, Ramakrishna S (2018). CD22-targeted CAR T cells induce remission in B-ALL that is naive or resistant to CD19-targeted CAR immunotherapy. Nat Med.

[131] Susanibar Adaniya SP, Cohen AD, Garfall AL (2019). Chimeric antigen receptor T cell immunotherapy for multiple myeloma: A review of current data and potential clinical applications. Am J Hematol.

[132] Raje N, Berdeja J, Lin Y, Siegel D, Jagannath S, Madduri D (2019). Anti-BCMA CAR T-cell therapy bb2121 in relapsed or refractory multiple myeloma. N Engl J Med.

[133] Eyquem J, Mansilla-Soto J, Giavridis T, van der Stegen SJ, Hamieh M, Cunanan KM (2017). Targeting a CAR to the TRAC locus with CRISPR/Cas9 enhances tumour rejection. Nature..

[134] Kim MY, Yu KR, Kenderian SS, Ruella M, Chen S, Shin TH (2018). Genetic Inactivation of CD33 in Hematopoietic Stem Cells to Enable CAR T Cell Immunotherapy for Acute Myeloid Leukemia. Cell.

[135] Stadtmauer EA, Fraietta JA, Davis MM, Cohen AD, Weber KL, Lancaster E, et al. CRISPR-engineered T cells in patients with refractory cancer. Science. 2020;367:eaba7365. 10.1126/science.aba7365 PMC11249135 32029687

[136] Sterner RM, Sakemura R, Cox MJ, Yang N, Khadka RH, Forsman CL (2019). GM-CSF inhibition reduces cytokine release syndrome and neuroinflammation but enhances CAR-T cell function in xenografts. Blood..

[137] Ohaegbulam KC, Assal A, Lazar-Molnar E, Yao Y, Zang X (2015). Human cancer immunotherapy with antibodies to the PD-1 and PD-L1 pathway. Trends Mol Med.

[138] Hamanishi J, Mandai M, Matsumura N, Abiko K, Baba T, Konishi I (2016). PD-1/PD-L1 blockade in cancer treatment: perspectives and issues. Int J Clin Oncol.

[139] Chen L, Han X (2015). Anti-PD-1/PD-L1 therapy of human cancer: past, present, and future. J Clin Invest.

[140] Dong H, Strome SE, Salomao DR, Tamura H, Hirano F, Flies DB (2002). Tumor-associated B7-H1 promotes T-cell apoptosis: a potential mechanism of immune evasion. Nat Med.

[141] Cyranoski D (2016). Chinese scientists to pioneer first human CRISPR trial. Nature..

[142] Cyranoski D (2016). CRISPR gene-editing tested in a person for the first time. Nature..

[143] Lu Y, Xue J, Deng T, Zhou X, Yu K, Deng L (2020). Safety and feasibility of CRISPR-edited T cells in patients with refractory non-small-cell lung cancer. Nat Med.

[144] Yi L, Li J (1866). CRISPR-Cas9 therapeutics in cancer: promising strategies and present challenges. Biochim Biophys Acta.

[145] Guo X, Jiang H, Shi B, Zhou M, Zhang H, Shi Z (2018). Disruption of PD-1 enhanced the anti-tumor activity of chimeric antigen receptor T cells against hepatocellular carcinoma. Front Pharmacol.

[146] He XY, Ren XH, Peng Y, Zhang JP, Ai SL, Liu BY (2020). Aptamer/peptide-functionalized genome-editing system for effective immune restoration through reversal of PD-L1-mediated Cancer immunosuppression. Adv Mater.

[147] Saayman S, Ali SA, Morris KV, Weinberg MS (2015). The therapeutic application of CRISPR/Cas9 technologies for HIV. Expert Opin Biol Ther.

[148] Gaglia MM, Munger K (2018). More than just oncogenes: mechanisms of tumorigenesis by human viruses. Curr Opin Virol.

[149] Mirabello L, Yeager M, Yu K, Clifford GM, Xiao Y, Zhu B (2017). HPV16 E7 Genetic Conservation Is Critical to Carcinogenesis. Cell.

[150] Kennedy EM, Kornepati AV, Goldstein M, Bogerd HP, Poling BC, Whisnant AW (2014). Inactivation of the human papillomavirus E6 or E7 gene in cervical carcinoma cells by using a bacterial CRISPR/Cas RNA-guided endonuclease. J Virol.

[151] Zhen S, Hua L, Takahashi Y, Narita S, Liu YH, Li Y (2014). In vitro and in vivo growth suppression of human papillomavirus 16-positive cervical cancer cells by CRISPR/Cas9. Biochem Biophys Res Commun.

[152] Lucifora J, Xia Y, Reisinger F, Zhang K, Stadler D, Cheng X (2014). Specific and nonhepatotoxic degradation of nuclear hepatitis B virus cccDNA. Science..

[153] Ramanan V, Shlomai A, Cox DB, Schwartz RE, Michailidis E, Bhatta A (2015). CRISPR/Cas9 cleavage of viral DNA efficiently suppresses hepatitis B virus. Sci Rep.

[154] Seeger C, Sohn JA (2014). Targeting hepatitis B virus with CRISPR/Cas9. Mol Ther Nucleic Acids..

[155] Seeger C, Sohn JA (2016). Complete Spectrum of CRISPR/Cas9-induced mutations on HBV cccDNA. Mol Ther.

[156] Dong C, Qu L, Wang H, Wei L, Dong Y, Xiong S (2015). Targeting hepatitis B virus cccDNA by CRISPR/Cas9 nuclease efficiently inhibits viral replication. Antivir Res.

[157] Feng J, Yang G, Liu Y, Gao Y, Zhao M, Bu Y (2019). LncRNA PCNAP1 modulates hepatitis B virus replication and enhances tumor growth of liver cancer. Theranostics..

[158] Liu Y, Qi X, Zeng Z, Wang L, Wang J, Zhang T (2017). CRISPR/Cas9-mediated p53 and Pten dual mutation accelerates hepatocarcinogenesis in adult hepatitis B virus transgenic mice. Sci Rep.

[159] Price AA, Sampson TR, Ratner HK, Grakoui A, Weiss DS (2015). Cas9-mediated targeting of viral RNA in eukaryotic cells. Proc Natl Acad Sci U S A.

[160] Wang J, Quake SR (2014). RNA-guided endonuclease provides a therapeutic strategy to cure latent herpesviridae infection. Proc Natl Acad Sci U S A.

[161] Yuen KS, Chan CP, Wong NM, Ho CH, Ho TH, Lei T (2015). CRISPR/Cas9-mediated genome editing of Epstein-Barr virus in human cells. J Gen Virol.

[162] Guo R, Jiang C, Zhang Y, Govande A, Trudeau SJ, Chen F (2020). MYC Controls the Epstein-Barr Virus Lytic Switch. Mol Cell.

[163] Lampreht Tratar U, Horvat S, Cemazar M (2018). Transgenic mouse models in Cancer research. Front Oncol.

[164] Day CP, Merlino G, Van Dyke T (2015). Preclinical mouse cancer models: a maze of opportunities and challenges. Cell..

[165] Huang J, Chen M, Whitley MJ, Kuo HC, Xu ES, Walens A (2017). Generation and comparison of CRISPR-Cas9 and Cre-mediated genetically engineered mouse models of sarcoma. Nat Commun.

[166] Blasco RB, Karaca E, Ambrogio C, Cheong TC, Karayol E, Minero VG (2014). Simple and rapid in vivo generation of chromosomal rearrangements using CRISPR/Cas9 technology. Cell Rep.

[167] Xue W, Chen S, Yin H, Tammela T, Papagiannakopoulos T, Joshi NS (2014). CRISPR-mediated direct mutation of cancer genes in the mouse liver. Nature..

[168] Byrne AT, Alférez DG, Amant F, Annibali D, Arribas J, Biankin AV (2017). Interrogating open issues in cancer precision medicine with patient-derived xenografts. Nat Rev Cancer.

[169] He D, Zhang J, Wu W, Yi N, He W, Lu P (2019). A novel immunodeficient rat model supports human lung cancer xenografts. FASEB J.

[170] Kaufman CK, Mosimann C, Fan ZP, Yang S, Thomas AJ, Ablain J (2016). A zebrafish melanoma model reveals emergence of neural crest identity during melanoma initiation. Science.

[171] Liu CJ, Xie L, Cui C, Chu M, Zhao HD, Yao L (2016). Beneficial roles of melanoma cell adhesion molecule in spinal cord transection recovery in adult zebrafish. J Neurochem.

[172] Weiss FU, Marques IJ, Woltering JM, Vlecken DH, Aghdassi A, Partecke LI (2009). Retinoic acid receptor antagonists inhibit miR-10a expression and block metastatic behavior of pancreatic cancer. Gastroenterology.

[173] Drabsch Y, He S, Zhang L, Snaar-Jagalska BE, ten Dijke P (2013). Transforming growth factor-β signalling controls human breast cancer metastasis in a zebrafish xenograft model. Breast Cancer Res.

[174] Zhang B, Shimada Y, Kuroyanagi J, Umemoto N, Nishimura Y, Tanaka T (2014). Quantitative phenotyping-based in vivo chemical screening in a zebrafish model of leukemia stem cell xenotransplantation. PLoS One.

[175] Yang XJ, Cui W, Gu A, Xu C, Yu SC, Li TT (2013). A novel zebrafish xenotransplantation model for study of glioma stem cell invasion. PLoS One.

[176] Moshal KS, Ferri-Lagneau KF, Haider J, Pardhanani P, Leung T (2011). Discriminating different cancer cells using a zebrafish in vivo assay. Cancers (Basel).

[177] Mayrhofer M, Mione M (2016). The toolbox for conditional Zebrafish Cancer models. Adv Exp Med Biol.

[178] Ablain J, Xu M, Rothschild H, Jordan RC, Mito JK, Daniels BH (2018). Human tumor genomics and zebrafish modeling identify SPRED1 loss as a driver of mucosal melanoma. Science..

[179] Dai F, Wu Y, Lu Y, An C, Zheng X, Dai L (2020). Crosstalk between RNA m6A modification and non-coding RNA contributes to Cancer growth and progression. Mol Ther Nucleic Acids.

[180] Chen XY, Zhang J, Zhu JS (2019). The role of m6A RNA methylation in human cancer. Mol Cancer.

[181] Lan Q, Liu PY, Haase J, Bell JL, Hüttelmaier S, Liu T (2019). The critical role of RNA m6A methylation in Cancer. Cancer Res.

[182] Liu XM, Zhou J, Mao Y, Ji Q, Qian SB (2019). Programmable RNA N6-methyladenosine editing by CRISPR-Cas9 conjugates. Nat Chem Biol.

[183] Wilson C, Chen PJ, Miao Z, Liu DR. Programmable m6A modification of cellular RNAs with a Cas13-directed methyltransferase. Nat Biotechnol. 2020;38:1431–40. 10.1038/s41587-020-0572-6 PMC7718427 32601430

[184] Prensner JR, Chinnaiyan AM (2011). The emergence of lncRNAs in cancer biology. Cancer Discov.

[185] Yao RW, Wang Y, Chen LL (2019). Cellular functions of long noncoding RNAs. Nat Cell Biol.

[186] Yi W, Li J, Zhu X, Wang X, Fan L, Sun W (2020). CRISPR-assisted detection of RNA-protein interactions in living cells. Nat Methods.

[187] Liu Q, Zhang H, Huang X (2020). Anti-CRISPR proteins targeting the CRISPR-Cas system enrich the toolkit for genetic engineering. FEBS J.

[188] Pawluk A, Davidson AR, Maxwell KL (2018). Anti-CRISPR: discovery, mechanism and function. Nat Rev Microbiol.

[189] Nakamura M, Srinivasan P, Chavez M, Carter MA, Dominguez AA, La Russa M (2019). Anti-CRISPR-mediated control of gene editing and synthetic circuits in eukaryotic cells. Nat Commun.

[190] He W, Wang H, Wei Y, Jiang Z, Tang Y, Chen Y, et al. GuidePro: a multi-source ensemble predictor for prioritizing sgRNAs in CRISPR/Cas9 protein knockouts. Bioinformatics. 2021;37:134–6. 10.1093/bioinformatics/btaa1068 PMC11025339 33394026

[191] Tasan I, Zhao H (2017). Targeting specificity of the CRISPR/Cas9 system. ACS Synth Biol.

[192] Tsai SQ, Zheng Z, Nguyen NT, Liebers M, Topkar VV, Thapar V (2015). GUIDE-seq enables genome-wide profiling of off-target cleavage by CRISPR-Cas nucleases. Nat Biotechnol.

[193] Tsai SQ, Nguyen NT, Malagon-Lopez J, Topkar VV, Aryee MJ, Joung JK (2017). CIRCLE-seq: a highly sensitive in vitro screen for genome-wide CRISPR-Cas9 nuclease off-targets. Nat Methods.

[194] Lazzarotto CR, Malinin NL, Li Y, Zhang R, Yang Y, Lee G (2020). CHANGE-seq reveals genetic and epigenetic effects on CRISPR-Cas9 genome-wide activity. Nat Biotechnol.

[195] Kosicki M, Tomberg K, Bradley A (2018). Repair of double-strand breaks induced by CRISPR-Cas9 leads to large deletions and complex rearrangements. Nat Biotechnol.

[196] Zhuo C, Zhang J, Lee JH, Jiao J, Cheng D, Liu L (2021). Spatiotemporal control of CRISPR/Cas9 gene editing. Signal Transduct Target Ther.

[197] Lee S, Kim YY, Ahn HJ (2021). Systemic delivery of CRISPR/Cas9 to hepatic tumors for cancer treatment using altered tropism of lentiviral vector. Biomaterials..

[198] Wei T, Cheng Q, Farbiak L, Anderson DG, Langer R, Siegwart DJ (2020). Delivery of tissue-targeted scalpels: opportunities and challenges for in vivo CRISPR/Cas-based genome editing. ACS Nano.

[199] Mashel TV, Tarakanchikova YV, Muslimov AR, Zyuzin MV, Timin AS, Lepik KV (2020). Overcoming the delivery problem for therapeutic genome editing: current status and perspective of non-viral methods. Biomaterials..

[200] Wang CS, Chang CH, Tzeng TY, Lin AM, Lo YL (2021). Gene-editing by CRISPR-Cas9 in combination with anthracycline therapy via tumor microenvironment-switchable, EGFR-targeted, and nucleus-directed nanoparticles for head and neck cancer suppression. Nanoscale Horiz.

[201] Charlesworth CT, Deshpande PS, Dever DP, Camarena J, Lemgart VT, Cromer MK (2019). Identification of preexisting adaptive immunity to Cas9 proteins in humans. Nat Med.

[202] Ihry RJ, Worringer KA, Salick MR, Frias E, Ho D, Theriault K (2018). p53 inhibits CRISPR-Cas9 engineering in human pluripotent stem cells. Nat Med.

[203] Haapaniemi E, Botla S, Persson J, Schmierer B, Taipale J (2018). CRISPR-Cas9 genome editing induces a p53-mediated DNA damage response. Nat Med.

[204] Enache OM, Rendo V, Abdusamad M, Lam D, Davison D, Pal S (2020). Author correction: Cas9 activates the p53 pathway and selects for p53-inactivating mutations. Nat Genet.

[205] Bowden AR, Morales-Juarez DA, Sczaniecka-Clift M, Agudo MM, Lukashchuk N, Thomas JC, et al. Parallel CRISPR-Cas9 screens clarify impacts of p53 on screen performance. Elife. 2020;9:e55325. 10.7554/eLife.55325 PMC7244323 32441252

[206] Cullot G, Boutin J, Toutain J, Prat F, Pennamen P, Rooryck C (2019). CRISPR-Cas9 genome editing induces megabase-scale chromosomal truncations. Nat Commun.

